# Visuomotor coordination on the road: Low-dimensional representations reveal adaptive, context-dependent reductions in the dimensionality of natural driving behavior

**DOI:** 10.1371/journal.pcbi.1014056

**Published:** 2026-10-23

**Authors:** John Madrid-Carvajal, Shadi Derakhshan, Peter König

**Affiliations:** 1 Institute of Cognitive Science, University of Osnabrück, Osnabrück, Germany; 2 Department of Neurophysiology and Pathophysiology, Center of Experimental Medicine, University Medical Center Hamburg-Eppendorf, Hamburg, Germany; Utrecht University: Universiteit Utrecht, NETHERLANDS, KINGDOM OF THE

## Abstract

The brain’s ability to transform complex, high-dimensional sensory and motor inputs into coordinated, goal-directed behavior remains a central challenge in neuroscience research. Current research suggests that behavior is generated through patterns within a low-dimensional structure. What visuomotor patterns underlie complex, unconstrained behaviors such as driving, and how stereotypical they are, remain poorly understood. Driving involves complex perception-action interactions, which we hypothesize are specific to the driver’s role and adapt to task demands. Here, we unfold these dynamics using an immersive virtual drive that recreates ten hazardous on-road events. We collected eye, head, and vehicle movements from participants assigned to either a manual or an automated driving condition. From the behavioral movements of 284 drivers, we constructed a common time-resolved behavioral state space based on collective variance, computed via principal component analysis across participants. The obtained low-dimensional representation thus captures generalized visuomotor coordination strategies. We then characterized the evolving low-dimensional structure with cosine similarity and various measures of effective dimensionality. We tested whether leading components contained condition-specific information using discriminant analysis. Throughout the entire drive, few components accounted for most of the variance in behavior, and effective dimensionality decreased reliably around critical events, indicating tighter coordination between perceptual and motor variables. During periods of low effective dimensionality, the contributions of eye, head, steering, and vehicle heading reorganized toward leading dimensions in a task-dependent manner. Across events, between-subject similarity was partially stable and stronger among manual drivers. This reorganization also made behaviors more distinct across driving conditions, enabling better classification of driving modes using only the first two components. These results show that naturalistic driving is supported by shared low-dimensional visuomotor coordination strategies that are flexibly reshaped by contextual demands, and provide a principled behavioral framework for linking neural manifolds, human driver models, and the design of adaptive automated vehicles.

## Author summary

Human behavior is complex. Driving, for instance, involves actively turning our head and eyes to see what matters most while translating those movements into appropriate actions with our hands and feet. Research using constrained repeated actions suggests that these seemingly complex behaviors are highly coordinated and can be described by a relatively small number of variables. We want to know whether this holds during natural, unconstrained tasks. We asked people to complete a virtual drive comprising ten hazards, such as animals and pedestrians on the road. Some participants drove the car, others sat in a self-driving car. We recorded where people looked, how they moved their heads, and steered the vehicle. Then, we used statistical methods to reduce the data’s dimensionality and looked for patterns that could explain all these signals together. We found that a small number of patterns were enough to describe behavior and became even simpler and more linked around hazards, differentiating automated from manual driving behavior. Our results suggest that even complex driving behavior follows a few basic coordination strategies specific to task demands. These basic dimensions contain important details that are unique to certain groups, ultimately helping us better understand and compare human behavior.

## Introduction

A major challenge when studying visuomotor coordination in large natural settings concerns the inherent complexity of actions. Ordinary everyday actions involve remarkably complex behaviors implicating many degrees of freedom (DoF). The DoF problem concerns the challenge of effectively controlling a system with so many variables that can move independently during natural behavior [1–3]. When walking, for instance, the central nervous system exerts control over many DoF within the musculoskeletal system, coordinating movement across multiple levels [4]. From the primary effectors (i.e., the muscles) to the resulting coordinated motion of the limbs, trunk, arms, head, and eyes, to the underlying bodily processes that occur along with them. Despite considerable progress in understanding how the central nervous system coordinates this high-dimensional movement system, the specific strategies that it utilizes to effectively control and solve the DoF problem during natural behavior remain an open question. Answering it requires a task that is complex and unconstrained enough to expose these strategies, yet structured enough for them to be compared across populations. We use driving as such a paradigm: our aim is not to characterize driving as such, but to use it as a window onto how complex natural behavior is organized.

A second source of behavioral complexity is variability, the observation that repeated executions of the same task rarely reproduce identical movements. Every single movement can be carried out in multiple ways, where even slight variations in body posture can lead to different outcomes [5,6]. It arises during motor preparation [7] and is likely linked to errors in the sensory estimates of the external world [8]. This variability has typically been studied separately within subjects and compared across subjects to assess similarities in the underlying mechanisms (i.e., stereotypy) [9–15]. Quantifying behavioral stereotypy (whether the central nervous system utilizes consistent strategies to organize behavior despite its extensive redundancy) requires developing solid frameworks that relate the multiscale and distributed dynamics inherent to behavior [16,17]. Equally important are methods to characterize the structure of behavior itself [18–23]. Notably, these methods have been predominantly applied to animal behavior. A key concept in this context is dimensionality, which quantifies the size of the subspace effectively used for behavior relative to the space of all possible movements [16,19,24]. Essentially, higher variability in behavior may reflect a higher dimensionality, indicating a broader range of directions in the movement space. Conversely, lower variability suggests movement is restricted to a narrower set of control strategies. Thus, understanding how the central nervous system produces coordinated behavior despite redundancy implies determining its dimensionality.

The difficulty of analyzing complex, variable behavior has led contemporary research to utilize dimensionality reduction techniques to reduce high-dimensional behavioral and neural data into a low-dimensional structure. Linear approaches such as principal component analysis (PCA) have proven particularly useful [25–30], yielding linear subspaces that may serve as local building blocks for describing more complex, nonlinear structures such as manifolds [31,32]. PCA reduces data complexity to a fewer number of orthogonal principal components (PCs) capturing key covariation patterns among the original variables; each PC is associated with an *eigenvalue* that quantifies the magnitude of the variance it captures [33–35]. The eigenvalue spectrum further allows for estimating the data’s effective dimensionality (ED) by quantifying how evenly variance is distributed across components, yielding an interpretable, information-driven measure of the equivalent number of independent dimensions needed to account for the overall pattern of covariation [24,36,37] (see Methods section). Low ED indicates tighter cross-variable coordination, making it a valuable and interpretable measure of how many dimensions behavior effectively occupies. In neuroscience, PCA has been used to directly relate behavioral data to its underlying neural activity, elucidating the types of transformations involved in the production and control of movement [38–41]. The core hypothesis suggests that, despite its many DoF and variability, behavior generation is constrained to a low-dimensional subspace or manifold that captures essential information for the planning, execution, and learning of movements [28,42,43]. These manifolds have successfully explained the organization of diverse behaviors, including locomotion [44–46] and reaching/grasping [25,27,47–49]. Moreover, such low-dimensional structural organization is consistently observed in non-human primates [50,51], mice [29,52,53], Caenorhabditis elegans [22], zebrafish [54], and birds [55]. A predominant aspect of the literature, however, is its reliance on stereotyped tasks and its primary focus on non-human species. Furthermore, the application of these methods to quantify complex natural human action is sparse, and the dimensionality of visuomotor coordination involving eye, hand, and head kinematics is understudied. This gap is underscored by recent research revealing a synergistic coupling of the eye-head and eye-hand systems during a pick-up and drop-off task [56]. Thus, applying dimensionality reduction to understand human visuomotor coordination in natural environments highlights a frontier for the study of human behavior. Driving is a particularly suitable paradigm for this purpose: unlike locomotion or grasping, it is a complex skill acquired during adulthood and trained in a common, systematic way, so that different individuals learn to solve the same visuomotor problem under comparable conditions. This makes driving well suited for asking whether individuals converge on shared coordination strategies, and for comparing those strategies across individuals and contexts.

Driving instantiates the demands outlined above in a compact way. It is a skill involving perceptually guided locomotion by means of a vehicle, initiated and motivated by the goal of reaching a specific destination and maintained by concurrent behaviors where *“the seeing and the doing are merged in the same experience”* [57]. The inherent unpredictability of real-world traffic poses a significant challenge for visuomotor systems, which must adapt rapidly to maintain safe and effective control. In this context, coordinated, skillful action involves overcoming superfluous DoF by transforming high-dimensional behavioral variables into common, synchronized features (i.e., motor synergies) that underlie the production of behavior [58,59]. Driving further allows the agent’s control role to be manipulated directly: when the vehicle drives itself, the perceptual demands of the situation largely persist while the motor demands are removed. Using driving in this way, the key question is therefore a general one: how many DoF does complex natural behavior truly require, which dimensions hold the relevant information, and how does this organization change when the contextual demands placed on the agent change?

Here, we translate the general motivation described above into three concrete questions about the organization of complex natural behavior, using naturalistic driving in virtual reality. First, whether behavior that is neither repeated nor experimentally constrained can still be described by a small number of coordinated dimensions. Second, whether this dimensionality is a fixed property of the behavior or is instead regulated by contextual task demands. And third, whether the resulting low-dimensional description is functionally meaningful, that is, whether it preserves information about the role of the agent, and whether its organization is stable across varying events. To address these questions, we analyzed eye-tracking and behavioral data recorded during a simulated virtual drive comparing manual control versus automated driving (SAE level 4) [60]. Crucially, we focused on variability across participants rather than on mere movement magnitudes, so that the recovered dimensions describe how different individuals solve the same visuomotor problem. By applying dimensionality reduction to the behavioral variables, we traced how these coordination patterns evolve over time and identified the variables contributing most to the dominant modes of variation. We then quantified the effective dimensionality of behavior around predefined critical events, tested whether the between-subject organization of the low-dimensional space is preserved across events, and employed discriminant analysis to quantify whether the resulting representation retains sufficient information to distinguish between driving conditions. Beyond confirming that behavior is low-dimensional, we show that it further compresses with task demands while the variables defining the leading dimensions reorganize, yielding a shared subspace in which context, individuals, and driving conditions can be compared directly.

## Results

### Research design

To understand the low-dimensional dynamics underlying complex natural behavior, we conducted a driving simulation using virtual reality (VR) (Fig 1A). To elicit naturalistic responses, the VR environment featured a real-life driving scene and ten critical events, involving the sudden appearance of predefined driving hazards, such as animals, pedestrians, and vehicles (Fig 1B and S1 Video). Participants were randomly assigned to a manual or an automated driving condition and remained seated behind the steering wheel (Fig 1C). During the drive, we recorded continuous eye, head, and vehicle-tracking data, yielding a multi-effector description of driving behavior (see Methods for details).

**Fig 1:**
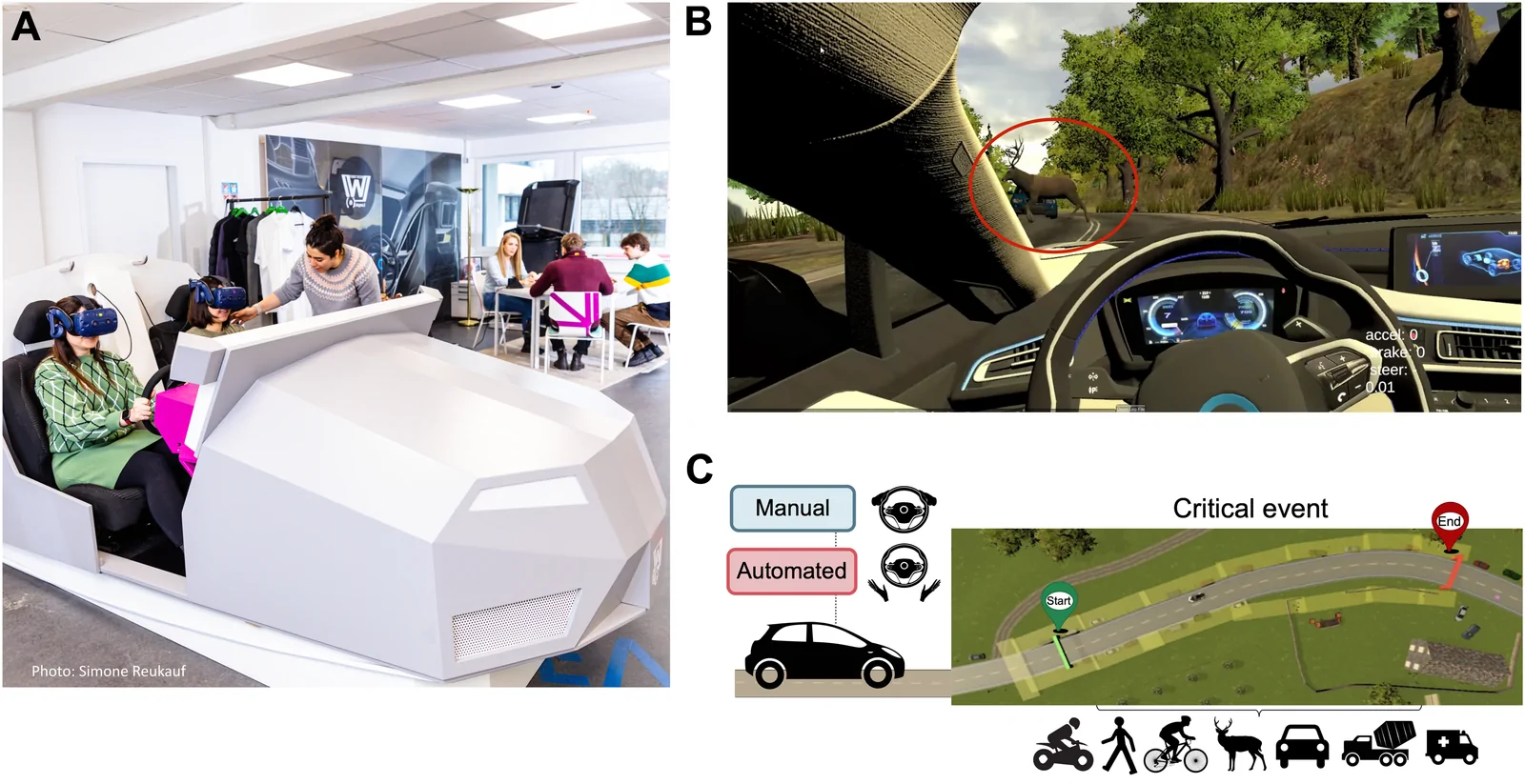
Experimental setup and virtual environment. **(A)** A photograph of the car mockup and devices used for the driving simulation, depicting a staged experimental setup with people wearing Head-Mounted Displays (HMD) (photo by Simone Reukauf). **(B)** Participants’ immersive view in virtual reality (VR), showing a reindeer dashing through the street–-the red circle was not visible to participants. **(C)** An aerial representation of the manual hands-on wheel (blue) and automated hands-off wheel (red) driving conditions in VR during a critical event from start to end.

To recover the structure of behavior and compare coordination strategies across participants, we applied a time-resolved principal component analysis (PCA) on 284 participants described by seven behavioral variables (Fig 2). Recordings were organized into a three-dimensional (3D) block structure (Fig 2A), from which we extracted participants × variables slices at each timepoint (Fig 2B). We then applied PCA to each segment [33–35], projecting the standardized variables onto a reduced set of principal components (PCs) that summarize how eye, head, car, and steering signals covary (Fig 2C). This approach yielded a continuous low-dimensional representation whose components and their explained variance we then tracked over time (see Methods).

**Fig 2:**
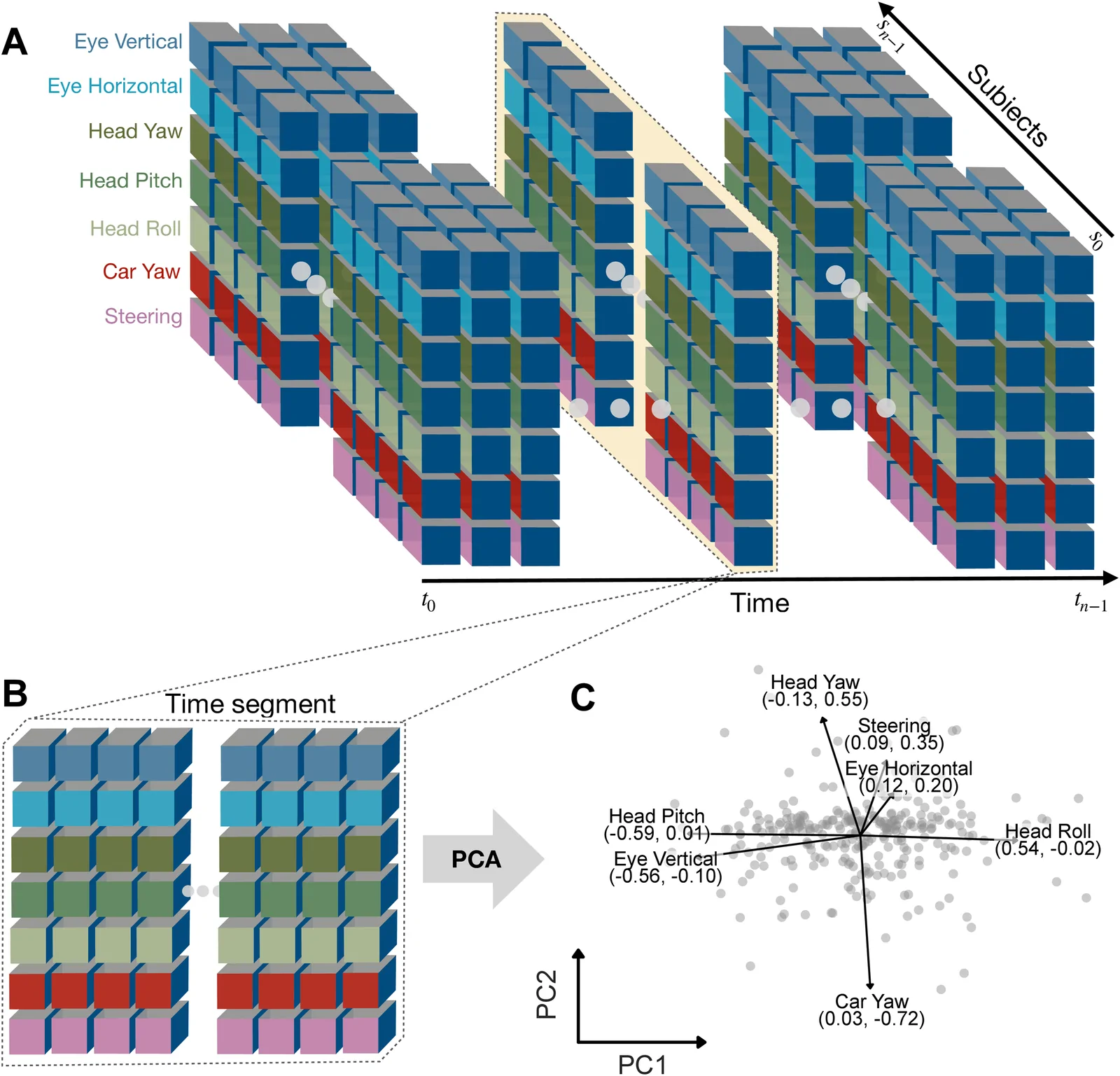
High-dimensional data segmentation and dimensionality reduction procedure. **(A)** An illustration of the multidimensional dataset composed of several behavioral parameters over time (x-axis), including eye, head, car, and steering movements, and color-coded for clarity. Each vertical block (y-axis) contains all behavioral data points for each subject (gray z-axis) within a specific timepoint from *t*_0_ to $t_{n-1}$. **(B)** A highlighted timepoint showing the extracted segment to which principal component analysis (PCA) is applied. **(C)** Resulting biplot after PCA, showing the distribution of the data after projecting it into the first two PCs (PC1 and PC2). Each gray circle in the biplot represents a subject, and the vectors indicate the contribution and correlation of each original variable in the reduced dimensional space; the larger the vector, the more it contributed to the corresponding PC.

### Temporal shifts in explained variance across principal components

Given the temporal nature of the data, we analyzed and compared several aspects of the PCA results. We first examined the temporal profile of eigenvalues and changes in explained variance. We then projected the behavioral variables onto the first two principal components (PCs) and assessed each variable’s representation within each PC throughout the drive. We concluded by calculating the data’s effective dimensionality (ED) from its eigenvalue spectrum using various ED estimators, and trained a logistic regression classifier to evaluate the separability of driving conditions using the scores for the first two PCs. This comprehensive analysis of the principal behavioral components extracted via PCA provides valuable insights into key aspects of driver behavior, facilitating comparisons of behavioral patterns between driving conditions.

After applying PCA, we first examined the explained variance associated with each behavioral PC over time to determine the dimensionality and dominant modes of variability in the data. The results show temporal shifts in explained variance across PCs: the first two PCs consistently captured a substantial proportion of the cumulative variance throughout the entire drive (PC1 *M* = 0.273, *SD* = 0.032; and PC2 *M* = 0.187, *SD* = 0.018), occasionally exceeding 50% of the cumulative variance (Fig 3). This pattern held throughout the drive, indicating that the data can be effectively represented by a low-dimensional structure characterized by the dominant influence of the PCs with the largest eigenvalues. This low-dimensional representation indicates that a large proportion of behavioral variability can be captured by the first two PCs, thereby reducing the complexity inherent in multivariate behavioral data. This suggests that the variability of behavior across drivers is surprisingly low-dimensional, with a few dominant modes accounting for how drivers differ in their core behavioral patterns.

**Fig 3:**
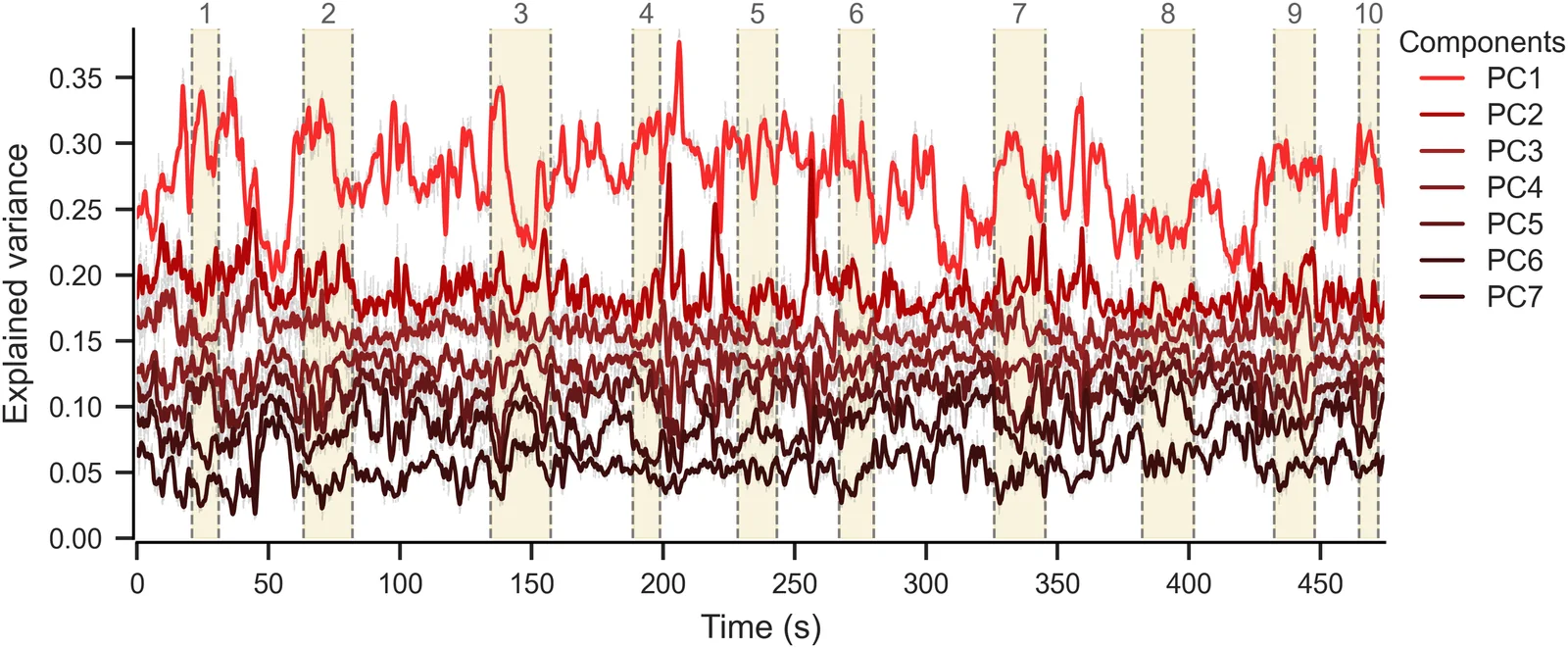
Temporal dynamics of the principal components’ explained variance over the duration of the drive. The thin gray lines denote the actual variance obtained from the dataset, serving as a reference for signal variability. Solid red lines depict the smoothed variance, calculated using a 50-sample rolling window (≈ 1 second), emphasizing trends in variance over time. Yellow windows represent the ten critical events, and dotted gray lines indicate their respective onsets and offsets, illustrating key moments that influence behavioral variance.

Task demands, however, fluctuate continuously during driving. The ten critical events mark moments of sudden and well-defined demand, allowing us to test whether the dimensionality of behavioral variability is fixed or changes with context. We therefore compared the variance structure within the ten critical event windows (151.6 s in total) with that of the remaining, non-critical driving periods (322.8 s). Outside critical events, the first two PCs jointly accounted for 45.7% of the total variance (PC1: *M* = 0.269, *SD* = 0.032; PC2: *M* = 0.187, *SD* = 0.019). Within critical events this share rose to 47.0% (PC1: *M* = 0.281, *SD* = 0.031; PC2: *M* = 0.189, *SD* = 0.017), an increase of 1.3 percentage points that was present in nine of the ten events. The first two PCs likewise exceeded 50% of the total variance during 19.6% of the time inside critical events, compared with 13.7% outside. This pattern is consistent with variance becoming more concentrated in the leading dimensions when demand rises, such that the data effectively occupy fewer dimensions and the behavioral variables become more tightly coupled in risk-related situations. Between-subject behavioral variability is therefore not only low-dimensional overall, but is further compressed for extended periods of time in context-specific situations. This contrast is coarse, however, as critical event windows differ substantially in length (7.3–22.9 s) and each is averaged as a single homogeneous block, so any change that is confined to part of an event is diluted by the remainder.

### Principal component variance reallocates around critical event onset

To quantify how drivers’ multivariate behavior reorganizes with task demands, we compared the distribution of variance before and after the onset of each of the ten critical events. Events were aligned to onset, and statistical analysis was performed within a fixed ± 5-second window, chosen to be shorter than the briefest event (7.3 s), to obtain comparable windows for all events. Across events, explained variance shifted modestly after onset (Fig 4). This redistribution was driven primarily by PC1, which captured a larger proportion of variance after onset (mean shifts upwards), indicating event-evoked reorganization. The remaining PCs captured less variance; PC2 through PC5’s variance remained stable between before and after onset windows, and PC6 and PC7’s variance decreased after onset (mean shifts downwards). Moreover, we found a significant difference between the before and after onset windows ($\chi^{2}(70,N=70)=23051.27$, *p* < 0.001). The pooled changes in variance across all event × PC pairs were small ($\hat{\Delta }=-0.002$, 95% confidence interval (CI) [-0.002, -0.002]), indicating a modest effect size. Since the total variance across before and after onset windows is 1, the variance difference indicates an increase in PC1 variance that was compensated by decreases in other PCs. Cochran’s Q redistribution test confirmed that these changes were not uniform across PCs but reflected a reallocation of variance toward the first two (*Q*(6, *P* = 7) = 7770.18, *p* < 0.001; *I*^2^ = 99.9%). Together, these results indicate a consistent, event-evoked reallocation of variance across events, consistent with stronger cross-variable coordination in a lower-dimensional space during hazard response.

**Fig 4:**
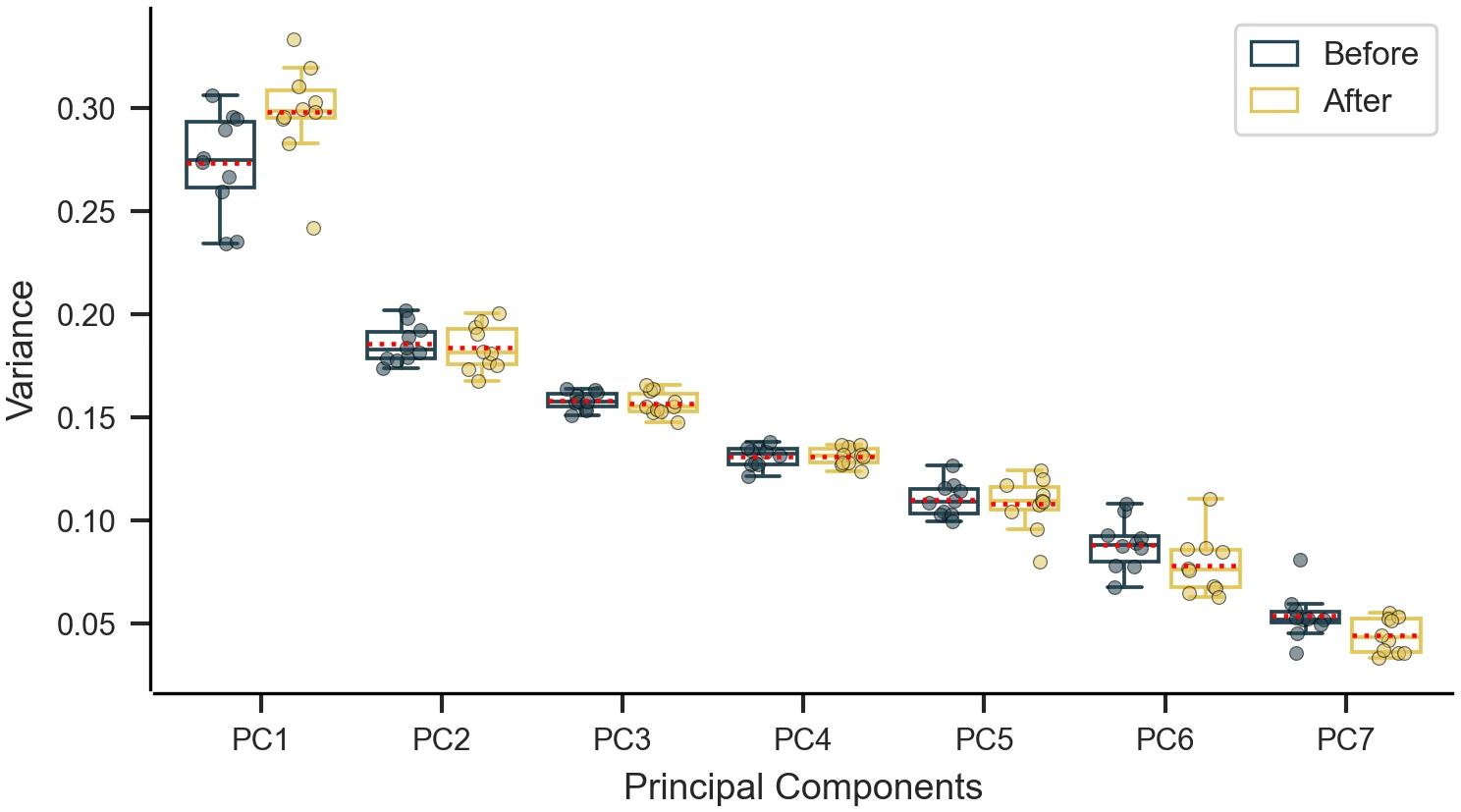
Distribution of variance around critical event onset for all ten events. For each PC, boxes represent the explained variance before and after critical event onset. All boxes show the interquartile range, the solid line is the median, the red dotted line marks the mean, and the circles denote the mean variance for each critical event. After onset, variance redistribution was driven primarily by PC1, with corresponding decreases in PC6-7’s variance.

### Tracing strategic timepoints of low and high dimensionality based on variance

So far, we have described broad and consistent patterns in the variance and dimensionality of driver behavior across multiple events. However, how behavioral variables relate to each other in the low-dimensional subspace remains unclear. To address this, we need to examine how variables project onto the first two PCs and how their contributions evolve over time. Because the full drive is a long time series, we narrow the focus to a single event to explore the temporal evolution of driving strategies. This allows a detailed examination of how variables interact over time, the mechanisms underlying shifts in dimensionality, and distinct behavioral adaptations that may be obscured when considering aggregated events.

Managing the uncertainty inherent in most traffic situations is challenging, yet drivers often navigate it successfully even in complex scenarios. Understanding how behavior is structured during such critical events is therefore crucial. For this analysis, we focus on a representative critical event (≈ 10s duration) that, upon event onset, depicts a left-hand curve with a hazard entering the roadway soon after (Fig 1B and S2 Video). Time-wise PCA on eye, head, car, and steering movements revealed a dynamic reallocation of variance over the course of the event (Fig 5). The variance of PC1, for instance, exhibits a clear temporal modulation, starting with a low initial magnitude at onset, followed by a sustained increase to maximum peak, and a subsequent decline (Fig 5A). We traced these strategic timepoints and found that from an initial 45% of cumulative variance explained at onset by the first two PCs (PC1 *eigenvalue* = 1.876, *SD* = 1.369, *variance* = 0.267; PC2 *eigenvalue* = 1.350, *SD* = 1.161, *variance* = 0.192), the system reached a moment of low effective dimensionality between 23.74 and 24.0 seconds. In that window, PC1 (*eigenvalue* = 2.384, *SD* = 1.544, *variance* = 0.339) and PC2 (*eigenvalue* = 1.393, *SD* = 1.180, *variance* = 0.198) jointly accounted for 54% of the cumulative variance. This increase in variance in the first two dimensions indicates the emergence of a more structured, low-dimensional behavioral pattern that coincided with clearer separation between automated and manual groups (Fig 5B). A subsequent drop in explained variance at around second 28.5 indicated a moment of high effective dimensionality (PC1 *eigenvalue* = 1.884, *SD* = 1.372, *variance* = 0.268; PC2 *eigenvalue* = 1.284, *SD* = 1.133, *variance* = 0.182), suggesting that the system became less structured, variables less coupled, so that more dimensions were required to explain behavior. This pattern is consistent with real-life driving, in which adaptable behavior becomes more or less structured and (condition-) specific through continuous transformations, despite the many DoF involved. These results show that complex behavior can be reduced to a low-dimensional space to identify moments of low and high effective dimensionality, illustrating the adaptive, time-varying structure of behavior around critical events.

**Fig 5:**
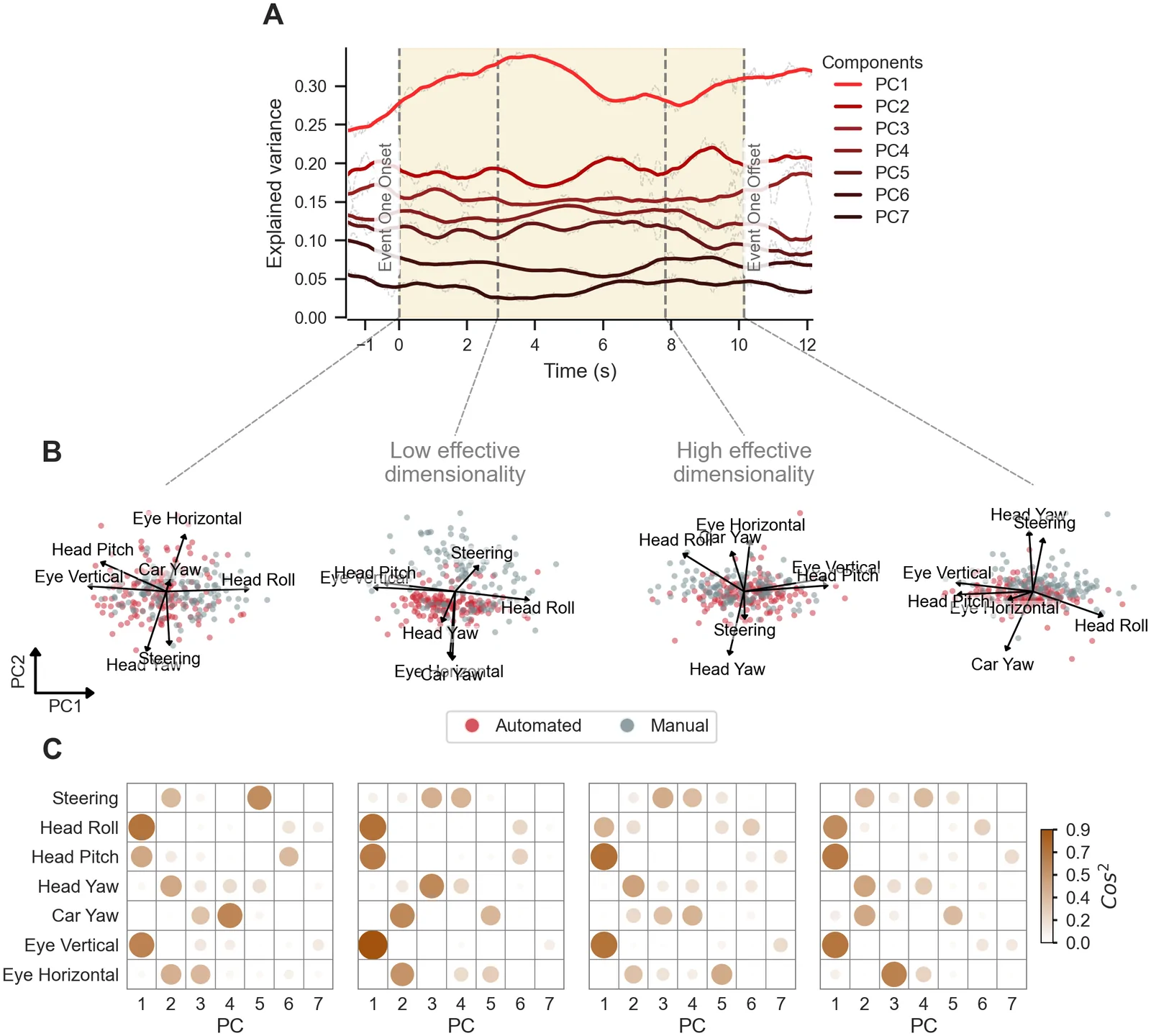
Principal component analysis (PCA) over the course of a critical event. **(A)** Temporal profile of eigenvalues for PC1 to PC7 before, during, and after a critical event. Dotted gray lines denote the variance for each PC, and solid lines depict the smoothed rolling mean (50-sample window). Vertical gray lines indicate critical event onset, offset, and the times of low and high effective dimensionality. **(B)** Biplot projections of behavioral variables onto the first two PCs at critical event onset (left), low effective dimensionality (middle-left), high effective dimensionality (middle-right), and critical event offset (right). Each point in the biplot represents a participant belonging to either a manual (blue) or an automated (red) driving condition. **(C)** Square cosine ($\cos^{2}$) depicting the quality of representation of all variables on the factor map. Each circle represents the quality of representation of each variable in the corresponding dimension. A high $\cos^{2}$ (dark brown) indicates good representation of the variable on the dimension. For any given variable, the sum of the $\cos^{2}$ across all dimensions is 1.

### Insights into visuomotor coordination strategies via biplots

We investigated the structure of these spaces of low and high effective dimensionality by projecting the behavioral variables onto PC1 and PC2 to visualize driver behavior in two dimensions (Fig 5B). This projection shows the relationship between the PCA scores and their loadings, providing a clear representation of how different behaviors couple and revealing correlations between the variables and the first two PCs (S3 Video). Projecting the data onto the strategic timepoints revealed different coordination strategies that unfold over time. At event onset, the biplot shows that manual and automated driving conditions occupy a shared region of the subspace. PC1 was primarily associated with a positive correlation with head roll (*r* = 0.843, *p* < 0.001) and a negative correlation with eye vertical (r=−0.799, *p* < 0.001) and head pitch (r=−0.667, *p* < 0.001), indicating that postural stability and vertical gaze control were the dominant factors in this dimension as lateral head tilt co-varies with opposite changes in vertical gaze and head movements. Minor but significant associations were also observed for eye horizontal (*r* = 0.191, *p* < 0.001) and head yaw (r=−0.203, *p* < 0.001). In contrast, PC2 was most strongly correlated with head yaw (r=−0.670, *p* < 0.001) and steering (r=−0.604, *p* < 0.001), establishing this principal behavior as an active steering orientation. This orientation was further defined by a strong, simultaneous opposing horizontal movement of eyes (*r* = 0.639, *p* < 0.001), head pitch (*r* = 0.327, *p* < 0.001), and to a lesser extent car yaw (*r* = 0.127, *p* < 0.05). At event onset, PC2 therefore captured a complex head-centric *“orient-and-scan”* behavior (head turning leads steering): drivers turn their heads and steer in one direction while their gaze scans in the opposite direction, likely to assess alternative paths or monitor the environment. The absence of clear condition separation in either dimension during event onset suggests a common baseline behavior.

These effects change over the course of the event. We therefore examined the timepoint after onset at which the cumulative variance explained by PC1 and PC2 peaked (*low effective dimensionality*; cumulative variance = 54%) (Fig 5A). At this timepoint, manual and automated driving conditions formed two clusters with partial overlap, highlighting both shared behavioral (PC1) and condition-specific (PC2) variability in the underlying dynamics (Fig 5B *– low effective dimensionality*). The analysis revealed a significant evolution in the shared behavioral strategy represented by PC1. At event onset, PC1 was primarily characterized by a compensatory relationship between head roll and vertical eye/head movements. As the event progresses, this pattern both strengthened and expanded. The underlying coupling represented by PC1 intensifies considerably: the negative correlations of eye vertical (r=−0.937, *p* < 0.001) and head pitch (r=−0.822, *p* < 0.001) with PC1 became much stronger than they were at onset, while head roll retained a comparably strong positive correlation (*r* = 0.852, *p* < 0.001). Compared to event onset, eye vertical and head pitch correlated more strongly with PC1 and with each other as indicated by the smaller angles between their vectors (i.e., they co-vary together), and both movement types were negatively correlated with head roll. Notably, the behavioral strategy represented by PC1 expands to incorporate other motor-related variables. It now showed modest but significant correlations with steering (*r* = 0.274, *p* < 0.001) and head yaw (r=−0.154, *p* < 0.05), indicating the emergence of a modest coupling that was absent at onset. At low effective dimensionality, steering and head yaw thus became more temporally coordinated with the shared behavioral dynamics captured by PC1. Importantly, this coupling reflects changes in the co-variation between these variables rather than an increase in the magnitude of their movements, as variables are normalized. This observed effect likely reflects a shift in how steering adjustments and head yaw movements are synchronized within this principal behavioral pattern, rather than an increase in the amplitude of their individual responses.

These after-onset coordination effects on how steering covaries with other behavioral variables became more evident in PC2 at the timepoint of low effective dimensionality. The behavioral dynamics captured by PC2 fundamentally reconfigured into a “coordinated turn execution” mode that separates the driving groups. In a substantial shift from the behavior at onset, PC2 became dominated by a unified coupling of gaze and vehicle heading, showing strong negative correlation with car yaw (r=−0.779, *p* < 0.001) and eye horizontal (r=−0.741, *p* < 0.001). The eyes now moved in the direction of the turn rather than against it, indicating a pattern in which drivers look smoothly into the turn as the vehicle turns. Concurrently, the contribution of head yaw decreased after onset (r=−0.354, *p* < 0.001), and now steering became a contrasting, positive correlate (*r* = 0.299, *p* < 0.001), reflecting a change in its covariation with the other variables. In contrast to the head-turning behavior seen at onset, heading now appears to rely less on turning the head and more on vehicle plus eye horizontal movements (vehicle leads). This strengthened coupling between car yaw, eye horizontal, and head yaw suggests that after onset, PC2 becomes an orientation-heading mode driven largely by horizontal movements, with steering acting as a secondary control variable that is particularly prominent for drivers in the manual condition.

This new dynamic, representing active, gaze-coupled control of the vehicle heading, is a characteristic signature of the manual driving condition, causing the driving groups to separate into distinct clusters along the PC2 axis (Fig 5B). Specifically, the biplot at the timepoint of low effective dimensionality shows that, in the normalized PC1–PC2 space, manual drivers differed primarily in how steering coordinates with the other variables, whereas drivers in the automated driving condition differed primarily in how horizontal movements coordinate with the rest. The partial overlap between the conditions suggests that, although certain variables contribute to condition separation, other factors (not captured by the included variables) lead to similarities between the conditions. Finally, at high effective dimensionality and at critical event offset, condition separability decreased, similarity scores spread across more dimensions, and behavioral effects became less consistent (Fig 5B and 5C and S1 Table). By jointly analyzing the variables’ contributions and their correlations with the first two PCs, we found a gradual emergence of after-onset steering covariation with other variables in PC1 that is expressed more prominently in PC2, leading to group separability. PC2 itself transitions from a head-centric orienting component at onset to a control-related vehicle/gaze-centric component after onset, indicating divergent strategies for vehicle heading control following event onset.

### Variables’ contributions redistribute towards leading dimensions after onset

Given the observed shift in variance after onset and the gradual reorganization of variable contributions toward an emerging low-dimensional structure, we assessed whether variables became more strongly represented in the leading dimensions. We calculated cosine similarity ($\cos^{2}$) to identify which variables contributed most to each dimension and to quantify the quality of their representation (Fig 5C and S3 Video). The similarity matrix indicated a clear pattern of consolidation. In the first dimension, we observed increased similarity for vertical head and eye movements from critical event onset (vertical eye $\cos^{2}=0.64$, head pitch $\cos^{2}=0.45$, head roll $\cos^{2}=0.71$) to the timepoint of low effective dimensionality (vertical eye $\cos^{2}=0.88$, head pitch $\cos^{2}=0.68$, head roll $\cos^{2}=0.73$). Notably, the contributions of vertical eye and head movements indicated a substantial and parallel growth, increasing by + 0.24 and + 0.23, respectively. This occurred alongside the already-dominant contribution of head roll, which served as a stable anchor for this dimension. The results also showed a modest similarity for steering in the first dimension that was absent at onset (steering $\cos^{2}=0.08$), consistent with the modest increase in correlation between this dimension and steering reported above. The implication of this coordinated convergence is the formation of a highly efficient eye-head-posture coupling, in which eye and head inputs merge to serve a single, prioritized function during critical events: maintaining vertical orientation.

A more dramatic transformation occurred in the second dimension. At event onset, PC2 was primarily defined by head-centric turning behavior (head yaw $\cos^{2}=0.45$, steering $\cos^{2}=0.37$). However, by the timepoint of low effective dimensionality, its contribution pattern had completely shifted. At this timepoint, the similarity of head yaw ($\cos^{2}=0.13$) and steering ($\cos^{2}=0.09$) with PC2 decreased drastically by −0.32 and −0.28, respectively. Meanwhile, horizontal movements of the car and eyes became dominant. Car yaw increased from $\cos^{2}=0.02$ at onset to $\cos^{2}=0.61$, and eye horizontal movements increased from $\cos^{2}=0.41$ to $\cos^{2}=0.55$, indicating a dynamic coupling of gaze to the vehicle’s motion. This contrast confirms a shift in the second dimension from a head-centric turning behavior to a vehicle-plus-eyes leading approach. This shift constitutes a functional handover, in which the underlying behavioral dynamics transition from representing the initiation of a turn to representing the execution and monitoring of the vehicle’s trajectory. Similar transformations can be observed at later stages of the event, such as subsequent timepoints of high effective dimensionality and at event offset, where variables’ similarity scores spread across more dimensions (Fig 5C), indicating that more dimensions are needed to achieve a comprehensive representation of the underlying behavior (S3 Video for a complete reconstruction of the similarity matrix over the course of the event). Crucially, the fact that variables’ similarity consolidates toward the leading dimensions precisely at the moment of low effective dimensionality demonstrates a temporary compression of the behavior into a more structured, parsimonious state.

### Effective dimensionality declines around critical event onset

Building on the single-event evidence showing that variables’ contributions concentrate in leading dimensions after critical event onset, we next sought to quantify the overall simplification of behavior around critical events and to determine whether this simplification generalizes across all event onsets. We hypothesized that the overall dimensionality of the data would be low and would be even lower after event onset. Because the raw eigenspectrum is a complex distribution of many eigenvalues rather than a single measure, we used established estimators to summarize the eigenspectrum into a single, interpretable index that reflects the data’s effective dimensionality (ED). ED quantifies how variance is distributed across dimensions and, consequently, the number of dimensions that are effectively used. Because it reduces the whole spectrum to one comparable number per timepoint, ED lets us track this simplification continuously and test it uniformly across all ten events. We quantified ED using four estimators (*n*_1_, *n*_2_, $n_{\infty }$, and $n_{C}$) within a ±5-second window around each critical event onset, excluding the onset sample itself from statistical analysis. Conceptually, these estimators quantify how “spread out” the variance is: if variance is concentrated in just a few dimensions, ED is low; if it is distributed across many dimensions, ED is high. The subscript for each estimator weights the contribution of dimensions differently: *n*_1_ provides a balanced assessment across all meaningful dimensions, *n*_2_ places greater weight on the dominant dimensions while discounting smaller ones, $n_{\infty }$ is the extreme case considering only the single largest dimension, and $n_{C}$ derives dimensionality by subtracting the variance of the eigenvalues from the total number of variables (see Methods section). Here, we report the results for all estimators and focus on *n*_2_ as it effectively discounts the large number of small eigenvalues often associated with increased sensor counts. This robustness makes *n*_2_ well suited for capturing the consolidation of behavior into a structured state without being overly influenced by noise.

To illustrate the effect of a critical event on dimensionality, we first examined the *n*_2_ trajectory for a single representative event. Before the event onset, ED was relatively stable, reaching a maximum of 6.2 dimensions. A sharp and rapid decline in ED began shortly before onset, reaching a minimum of 4.4 dimensions after onset (Fig 6A); a drop of almost 2 effective dimensions. We therefore asked whether this behavioral simplification is systematic across all critical events. The results revealed a clear and consistent pattern of sustained decrease in ED after onset across events (Fig 6B). While individual event trajectories of ED showed natural variability, they almost consistently drop below their before onset baseline. The mean trajectory (black line) confirmed a clear tendency for ED to decline immediately after onset, moving from a before onset average of about 5.8 dimensions to a sustained lower ED after onset. This trend was validated by strong statistical evidence. Event-level chi-squared tests revealed a significant reduction in *n*_2_ between the before and after onset windows $\chi^{2}(10,N=10)=8025.01$, *p* < 0.001, with an inverse-variance-weighted mean difference of $\hat{\Delta }=-0.275$ (95% confidence interval (CI) [−0.285, −0.266]), indicating consistent lower ED after onset across events. Furthermore, this event-evoked reduction in ED after onset was consistent across all four metrics (Holm-adjusted *p* < 0.001). Comparable effects were found for *n*_1_, $\chi^{2}(10,N=10)=6269.60$, *p* < 0.001, $\hat{\Delta }=-0.158$ (95% CI [−0.164, −0.151]), and $n_{\infty }$ $\chi^{2}(10,N=10)=9493.14$, *p* < 0.001, $\hat{\Delta }=-0.314$ (95% CI [−0.323, −0.305]). For $n_{C}$, the effect was smaller but still moderate, $\hat{\Delta }=-0.050$ (95% CI [−0.051, −0.048]), and the global before and after difference remained highly significant, $\chi^{2}(10,N=10)=7281.28$, *p* < 0.001. These results demonstrate that the simplification of behavior observed in a single event generalizes across events as a robust, stereotyped response in which the system consistently reduces complexity to navigate critical situations.

**Fig 6:**
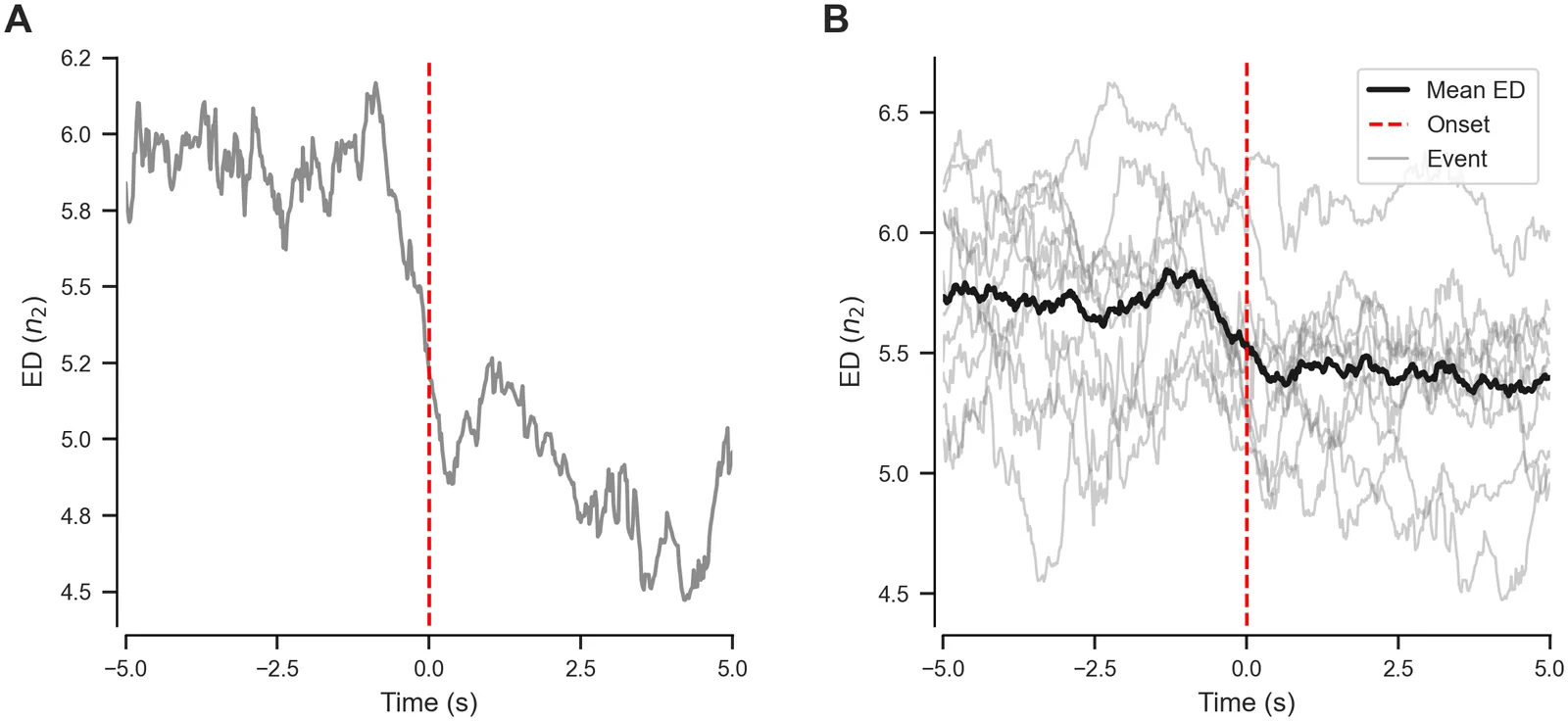
Critical events induce a lower-dimensional behavioral state. **(A)** The *n*_2_ effective dimensionality trajectory for a single, representative event within a ± 5-second window around onset. The ED is relatively stable early in the window and begins to decline shortly before onset (*t* = 0, red dashed line), then exhibits a sharp and rapid drop, illustrating a clear simplification of behavior. **(B)** This simplification proves systematic after aligning the ED trajectories for all ten critical events. Individual event trajectories (light gray lines) show natural variability but a consistent tendency to decrease after onset. The mean trajectory (thick black line) reveals a significant and sustained reduction in ED, a trend confirmed by an event-level chi-square test across several ED metrics comparing the before and after onset periods (Holm-adjusted *p* < 0.001).

### Between-subject similarity in low-dimensional space is partially stable across critical events

The preceding analysis established that behavior reliably compresses around critical events. This compression, however, does not guarantee that the low-dimensional space is organized in the same way from one event to the next, as two events could collapse onto an equally low-dimensional subspace while arranging drivers differently. We therefore asked whether the subspace of minimal behavior for one event, that is, the between-subject similarity that defines who resembles whom, is itself preserved across the ten hazards. At each event’s low effective dimensionality timepoint $t_{e}^{*}$, we quantified the relative positions of the identity-matched participants and compared them across events using second-order representational similarity analysis (S1 Fig; see Methods). Across the pooled population sample, all 45 event pairs were positively and significantly correlated (all *p* < 0.001, false discovery rate (FDR)-adjusted), revealing a statistically reliable component of inter-individual similarity shared across events (Fig 7A). The stability (*S*) of this shared organization, given by the mean second-order correlation across events, was nonetheless modest in magnitude (*S* = 0.28; 95% CI [0.25, 0.33]; pairwise range 0.16–0.46) and robust to the eigenvalue scaling of the subspace, persisting when distances were computed on shape-only (whitened) coordinates (*S* = 0.25; 95% CI [0.23, 0.29]). Thus the pooled analysis revealed a reliable but modest shared organization that was genuinely present yet unevenly distributed across events.

**Fig 7:**
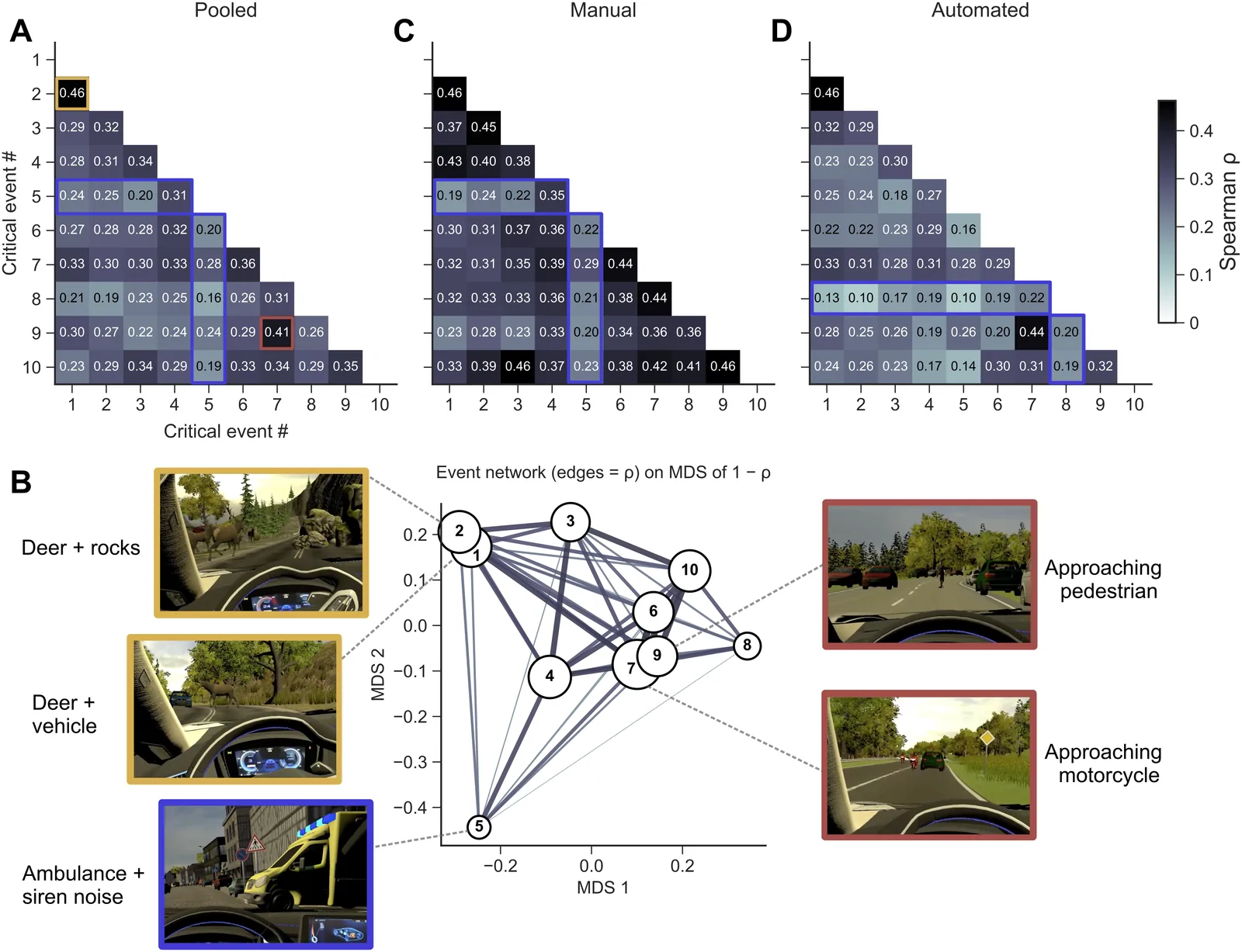
Cross-event stability of between-subject similarity in the minimal PC1–PC2 subspace. Each cell in the heatmaps is the Spearman correlation (ρ) between two events’ subject × subject Euclidean distance matrices (i.e., representational dissimilarity matrices (RDMs)), computed at the event’s low effective dimensionality timepoint $t_{e}^{*}$ (window-averaged ±0.5 s). **(A)** Events’ correlations pooled across all identity-matched participants. All 45 event pairs were significant (subject-level Mantel test, FDR-adjusted). **(B)** Multidimensional scaling (MDS) embedding of events from 1−ρ using the primary pooled correlates of RDMs across events, where nearby points indicate events with more similar between-subject structure. **(C)** Events’ correlations for manual group and **(D)** Events’ correlations for automated group. Blue outlines highlight the most dissimilar event for each group.

Events contributed differently to this shared structure. Consistent with substantial event-specific characteristics, the pooled population matrix shows that the between-subject similarity, although positive, varied across events (Fig 7A). Event five had the lowest mean second-order correlation with the remaining events ($\bar{\rho }=0.23$), compared to a maximum of $\bar{\rho }=0.33$ for event seven. These differences between events were clearer in the two-dimensional embedding obtained via multidimensional scaling (MDS), which places events in a plane so that their pairwise distances approximate the representational dissimilarities (1−ρ; see Methods), making events with more similar between-subject structure lie closer together (Fig 7B). In the event network, event five stood apart as representationally distinct from all other events, whereas events one and two (ρ=0.46), and events seven and nine (ρ=0.41), formed neighboring pairs being the strongest correlations observed. Interestingly, event five was the only event that included a loud noise (ambulance siren), events one and two both involved animal hazards, and events seven and nine involved an approaching hazard. Together, these observed correlations seem to reflect or be driven by both context-specific characteristics shared across events and the similarity of the drivers’ behavior around hazards.

Finally, the shared low-dimensional behavioral structure across events also emerged within each driving condition. All 45/45 event pairs were positively and significantly correlated (*p* < 0.001, FDR-adjusted) in both groups (Fig 7C and 7D). On average, second-order correlation across all events was stronger among the manual (*S* = 0.34, 95% CI [0.29, 0.42]) than the automated group (*S* = 0.25, 95% CI [0.21, 0.32]) and a subject-label permutation test revealed that this difference was statistically reliable (ΔS=0.09, *p* = 0.035). The least-similar event also differed by condition: averaging each event’s mean second-order correlation with the remaining events, event five remained the most dissimilar among manual group ($\bar{\rho }=0.24$) (Fig 7C blue outline), whereas event eight was the most dissimilar among automated group ($\bar{\rho }=0.17$), with event five only slightly higher ($\bar{\rho }=0.21$) (Fig 7D blue outline). Notably, more event pairs showed a higher similarity in the manual than in the automated driving condition. Together, these results indicate that the low-dimensional organization of individuals is *partially* stereotyped across events: whereas the degree of compression documented above recurs as a robust, stereotyped response, the arrangement of drivers within the compressed space combines a consistent, statistically reliable component of who is similar to whom with a substantial event-specific one. This partial stability aligns with the context-dependent nature of the compression itself.

### Driving conditions have distinct low-dimensional representations within PC1-PC2

After having established the low-dimensional nature of behavior, we next tested whether this simplified PC1-PC2 subspace of multi-signal behavior was functionally meaningful. Specifically, we hypothesized that it would capture distinct behavioral signatures of manual versus automated driving, allowing for separation between the two conditions. To achieve this, we assessed both the geometric separability of the conditions and their predictive power using a time-resolved discriminant and classification approach. First, we examined the geometric distance between the two conditions within the PC1-PC2 space. For a representative event (the same left-hand-curve event described above; S2 Video), the discriminant scores analysis shows consistent separation between manual (*M* = 0.43, *SD* = 0.14) and automated driving groups (M=−0.43, *SD* = 0.14) with transient windows where separability increases early after critical event onset (Fig 8A). The centroid distance between the groups confirmed this separation, averaging a centroid distance of 0.86 (*SD* = 0.28; 95% CI: [0.58, 1.15]), indicating a statistically robust separation across time that strengthens during certain periods (Fig 8B). This effect was sustained across events with an average centroid distance of 0.80 (*SD* = 0.21; 95% CI: [0.69, 0.93]) (Table 1). This geometric analysis provides evidence that the low-dimensional representations of manual and automated driving conditions captured by the primary PC1-PC2 behavioral subspace correspond to robustly separable states.

**Fig 8:**
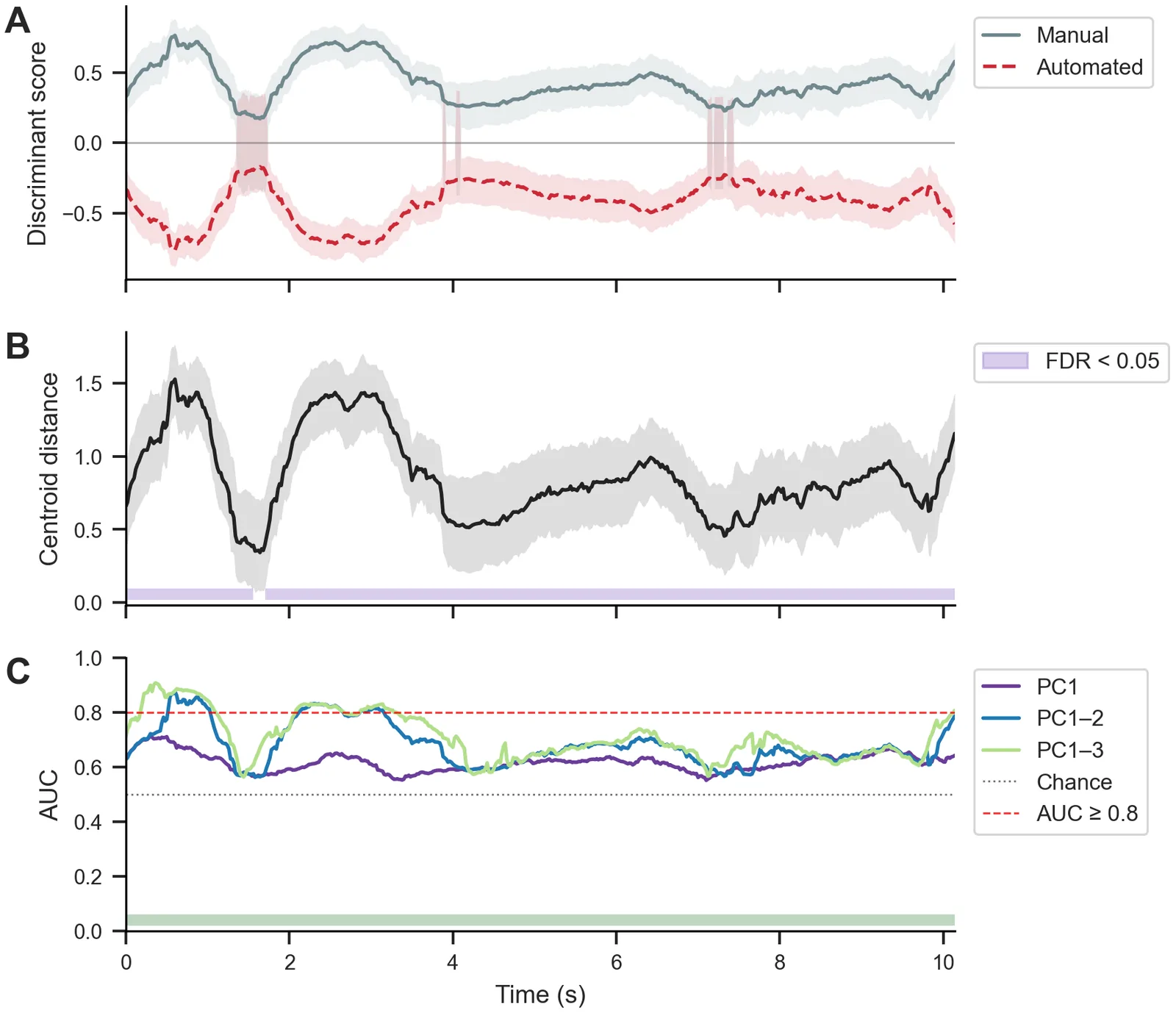
Driving conditions have distinct low-dimensional representations in PC1-PC2 subspace. Three panels showing that the first two principal components (PC1-PC2) of behavior are sufficient to robustly separate manual and automated driving conditions. **(A)** Discriminant scores over time for a representative event show a clear separation between the manual (solid blue) and automated (dashed red) conditions. Shaded areas represent 95% bootstrap CIs (resampling participants within each group at each time **t)**, indicating minimal overlap between the two conditions. **(B)** The geometric distance between the two group centroids ($D=\|c_{\text{Manual}}-c_{\text{Auto}}\|$) in the PC1-PC2 space over time with 95% bootstrap CIs (class-stratified resampling at fixed **t)**. The purple bar at the bottom highlights timepoints where this distance is statistically significant as obtained from subject-level label permutations (FDR-adjusted). **(C)** Predictive power of the PC subspace as a function of the number of components retained, measured by the area under the curve (AUC) of a stratified 5-fold logistic regression classifier with obtained out-of-fold probabilities. AUC time courses are shown for PC1 alone (purple), PC1-PC2 (blue), and PC1-PC2-PC3 (green). The classifier consistently exceeds chance performance (dotted gray line, AUC = 0.5), with several timepoints achieving high accuracy (dashed red line, AUC ≥0.8). Adding a third component yielded only a marginal improvement over two components. The green bar at the bottom indicates FDR-adjusted significance from subject-level label permutations applied to the PC1-PC2 model.

**Table 1:** Across-event summary (mean ± SD, 95% bootstrap CI across events).

Metric	Mean	SD	CI_low_	CI_high_	*n*_events_
Manual disc. score	0.40	0.10	0.34	0.46	10
Automated disc. score	-0.40	0.10	-0.46	-0.34	10
|Manual−Automated| disc. difference	0.80	0.21	0.69	0.93	10
Centroid distance	0.80	0.21	0.69	0.93	10
AUC at per-event max	0.87	0.07	0.83	0.91	10
Precision@0.5 at max AUC	0.82	0.08	0.77	0.87	10
Recall@0.5 at max AUC	0.77	0.10	0.71	0.83	10
F1@0.5 at max AUC	0.80	0.09	0.74	0.85	10
Max F1 at max AUC	0.82	0.07	0.78	0.87	10
AUC (event means)	0.67	0.05	0.65	0.71	10
Precision@0.5 → event means	0.64	0.05	0.61	0.67	10
Recall@0.5 → event means	0.52	0.08	0.47	0.56	10
F1@0.5 → event means	0.57	0.07	0.53	0.61	10
Max F1 → event means	0.68	0.03	0.66	0.70	10

Second, we assessed whether the PC1-PC2 subspace retains enough condition-specific information for a classifier to predict the driving condition. For the same representative event, the classifier performance reached a maximum area under the curve (AUC) score of 0.87 (precision@0.5 = 0.80, recall@0.5 = 0.77), demonstrating the existence of transient windows of strong separability (Fig 8C). While the average performance over the entire event was lower (AUC = 0.68, *SD* = 0.07), the classifier’s discriminatory power remained significantly above chance (Fig 8C bottom green highlight). Across all events (*N* = 10), the classifier consistently performed above chance with an average AUC of 0.67 (*SD* = 0.05; 95% CI: [0.64, 0.70]). Crucially, when examining the timepoint of maximum discriminability within each event, the classifier achieved a mean peak AUC of 0.87 (*SD* = 0.07; 95% CI: [0.82, 0.91]). This indicates that, while the exact timing may vary, each critical event contains a clear, transient window in which the behavioral signatures of the two driving conditions are highly distinct in the PC1-PC2 subspace. To verify the sufficiency of two components, we performed a component-sweep ablation by training the same cross-validated classifier on PC1 alone, PC1-PC2, and PC1-PC2-PC3 (Fig 8C). For the representative event, mean AUC increased from 0.61 (PC1) to 0.68 (PC1-PC2, ΔAUC=+0.07), while adding a third component yielded only a marginal gain (ΔAUC=+0.03). This confirms that the first two PCs capture enough condition-discriminative information and that adding a third component contributes little. Together, these geometric and predictive analyses demonstrate that major behavioral differences between manual and automated driving are captured within just the first two PCs. These findings support the idea that the low-dimensional subspace is not merely a statistical summary but a functionally relevant representation of driver behavior.

## Discussion

This study aimed to examine the low-dimensional dynamics of complex, natural behavior. Naturalistic driving in virtual reality revealed that a small set of latent dimensions captures most of the variance in eye, head, and vehicle behavior, and that this effective dimensionality reduces further around hazardous events. Throughout the drive, the first two principal components (*eigenbehaviors*) consistently captured most of the variance, frequently exceeding half of the total variance. Established estimators of effective dimensionality further supported the idea that only a few dimensions were effectively used [24,36]. Around critical events, variance reallocates modestly toward PC1 (with smaller changes in PC2), and effective dimensionality drops. Consequently, the contributions of the behavioral variables reorganized: early after onset, vertical eye movements and head pitch dominated alongside head roll, whereas slightly later, horizontal eye and vehicle heading became more pronounced and the coordination between steering and the other behavioral variables became a defining feature of the low-dimensional structure, particularly for manual drivers. Within this compressed subspace, manual and automated driving groups occupied overlapping but reliably separable regions. Between-subject similarity within this subspace was partially stable across events, and more so among manual drivers. Discriminant and logistic regression classification analyses using the first two principal components supported these findings, demonstrating that both driving conditions occupied clear, separate regions in the PC1-PC2 subspace, with significantly above-chance classification accuracy and strong separability during transient time windows. These analyses build on one another: only once a low-dimensional description holds, it is meaningful to ask what its dimensionality is and whether it varies with context, what the leading dimensions are composed of at those moments, whether between-subject similarity recurs across events, and whether the subspace still distinguishes driving conditions. Together, these findings show that complex natural behavior is not merely low-dimensional, but that its dimensionality is actively regulated: the number of effective dimensions and the variables that constitute them change independently, contracting in anticipation of task demand while re-composing to serve the control problem at hand. The consequence is a common coordinate system in which the same behavior can be compared across contexts, individuals, and control roles, with manual and automated driving occupying one shared subspace yet leaving reliably distinct signatures within it.

These results provide a naturalistic, task-level perspective on the long-standing degrees-of-freedom problem [1,3,6]. Previous work on motor synergies has shown that coordinated action in grasping and locomotion can be described in terms of low-dimensional modules [41,45–49], and that neural population activity evolves on low-dimensional manifolds supporting movement generation [29,38,39,51]. In line with these frameworks, treating behavior as trajectories on a structured manifold, the observed reduction of effective dimensionality around critical events suggests that the system temporarily collapses onto a smaller set of coordinated modes that channel visuomotor variability into task-relevant degrees of freedom. Two consequences follow. First, because compression begins before the hazard has fully unfolded, effective dimensionality seems to index anticipatory control rather than a reaction to an event, so the timing of compression becomes a candidate readout of when an agent commits to a course of action. Second, because the number of dimensions remained small while their composition changed, a low-dimensional description does not amount to a loss of behavioral detail but to a change of basis: the informative signal lies in which variables enter the leading dimensions, and it is this composition, rather than the count, that distinguishes an agent controlling the vehicle from one merely monitoring it. This extends dimensionality reduction approaches that have been predominantly applied to animal behavior and repetitive human tasks [18–23,54] to complex, multi-effector human behavior. Driving is informative in this regard precisely because it is learned late and systematically: unlike locomotion, which develops in infancy, adults acquire driving through comparable instruction and licensing, so convergence in coordination strategies across drivers is more plausibly attributable to shared constraints on control than to a shared developmental trajectory. Taken together, these findings indicate that the driver-vehicle system forms a functional unit [61] whose behavior can be understood in terms of a small set of context-sensitive coordination patterns.

Analyzing such high-dimensional data requires specific choices regarding how the multidimensional data structure (e.g., participants×features×time) is decomposed, as the matricization strategy dictates the biological interpretation of the resulting manifold. Here, we applied a time-resolved, cross-sectional strategy: at each timepoint we stacked participants to form a subjects×features matrix. Consequently, the PCA extracts directions of maximal inter-individual covariation, yielding a group-level manifold that captures shared visuomotor coordination strategies and enables direct comparison of behavioral modes across individuals [15]. This contrasts with approaches that leverage repeated trial structure, applying PCA across repetitions of similar actions within individual subjects [56]. Such an approach characterizes intra-individual trial-to-trial variability, highlighting how the eye, head, and hand couple synergistically to support consistent action planning despite motor noise. While such “repetition slicing” summarizes the consistency of motor execution, our cross-sectional approach captures the structural variability of coordination strategies across the population. This distinction is fundamental for interpretation: because our principal components are defined by between-participant variance, separation in the manifold reflects differences in style (e.g., how steering covaries with gaze and head orientation) rather than trial-by-trial fluctuations in execution. Thus, our approach reveals how different drivers solve the same motor problem, rather than how a single driver stabilizes a movement across attempts.

Our results speak directly to the three questions raised above. Visuomotor coordination during natural driving is constrained to a low-dimensional manifold, its dimensionality is regulated rather than fixed, and the compressed description remains functionally informative. The variance explained by the first two principal components fluctuates throughout the drive, occasionally exceeding 50% of the total variance. This aligns with previous findings indicating that manifolds for unconstrained behaviors exhibit slightly higher dimensionality than those of constrained behavior, with low-dimensional manifolds carrying task-relevant information [26]. The variability of this task-relevant information has been shown to be smaller than that of task-irrelevant information [62]. Consistent with a shift toward such a task-relevant, low-dimensional regime, we observed two complementary patterns around critical event onsets: the reorganization of variables’ contributions toward the leading dimensions and a consistent, significant decrease in effective dimensionality across all events. These patterns are reflected in the variance of individual components. PC1 captured a larger share of variance after onset, compensated by trailing components (PC6-PC7), while PC2-PC5 remained relatively stable. Yet a stable share of variance does not imply a static composition. In the representative event, PC2 shifted from head-centric turning behavior to a coupling of gaze with vehicle heading. Compression thus reflects a steeper eigenvalue spectrum and a change in which variables define the leading dimensions. Notably, the decrease in effective dimensionality often began shortly before the event onset, suggesting an anticipatory, preparatory reorganization of behavior.

Furthermore, the obtained subspace carries condition-specific information that differentiates between driving groups. Manual driving was characterized by steering-dominant coordination patterns within the low-dimensional space. In contrast, automated driving was defined by the covariance structure of horizontal gaze and car heading, consistent with drivers monitoring the environment and the automated system rather than controlling the vehicle. This contrast is partly expected since steering carried between-participant variance almost exclusively in the manual condition. The informative result is thus not that steering separates the groups, but that in both groups gaze couples with the direction of the car: actively, together with steering in the manual condition, and passively, in the absence of steering control in the automated condition. That is, automated drivers still gaze toward where the car is heading even though they do not steer it. This observation aligns with findings that the underlying visuomotor coordination for steering persists even when vehicle control is automated [63]. The discriminant analysis and classification performance demonstrate that a small number of latent dimensions is sufficient to capture these differences, suggesting that the obtained manifold reflects meaningful functional structure. Together, these results indicate that adaptive, context-dependent reductions in dimensionality are a hallmark of skilled behavior, rather than simply by-products of biomechanical constraints.

These results support a conceptual link between low-dimensional behavior and the design of automated driving systems. First, the condition signatures within the PC1-PC2 subspace point to candidate features for driver monitoring systems that distinguish active control from monitoring. Because steering is by definition absent under automation, the more informative feature is the coupling of horizontal gaze with vehicle heading, which persisted without steering input and could indicate whether a driver who is not controlling the vehicle still monitors its trajectory, remaining in the loop [64,65]. Second, the transient compression of behavior around critical events, together with the transient windows of strongest separability between driving conditions, suggests that driver state is most readable in brief, event-locked windows, which could help a system determine whether a driver is engaged or not and thus potentially ready to intervene [66]. However, such signatures should not be used in isolation. Effective dimensionality also decreased during environmentally constrained segments, so these signatures would need to be combined with information about the traffic scene. Finally, participants in both driving conditions sat behind the steering wheel (SAE level 4) [60]. This may itself signal that intervention could be required and thereby keep drivers alert, which could explain why gaze remained coupled to the vehicle’s heading. These signatures should therefore not be assumed to hold for fully automated (SAE level 5) driving where no driving-related intervention is possible.

The issue of developing methodologies to capture and analyze the structure of complex human behavior in natural settings remains relevant. Despite the large sample and rich behavioral dataset, certain limitations constrain the extent to which our findings can address broader questions regarding the dimensionality of visuomotor behavior. First, although principal component analysis (PCA) has proven useful for uncovering low-dimensional structures across behavioral and neural data [12,32,41,51], the debate persists over whether linear or more complex nonlinear dimensionality reduction techniques are better suited for capturing the underlying dynamics [27,67]. Indeed, independent time-wise PCA analyses ignore temporal dependencies in the behavioral signals, treating each time slice as statistically independent. This choice allows a straightforward, interpretable characterization of moment-to-moment coordination patterns, but fails to capture how coordination evolves through time and its underlying continuous functional form. To understand these spatial and temporal structures, future work could examine more temporally aware methods, such as functional PCA [68–70] and other nonlinear methods [26,71–73]. These methods could, for instance, help elucidate how task-relevant components are modulated over time and by task demands.

Second, although a representative set of eye, head, and vehicle variables was used, additional factors might prove valuable in investigating the effective dimensionality of driving behavior. These might include pedal forces, body postures, or variables explicitly related to decision-making. These variables likely contribute additional information and could help explain residual variability and the partial overlap among driving groups. Here, however, we restricted the primary analysis to signals that directly register eye–head–hand–vehicle angular coordination (i.e., the perception–action loop). Thus, variables describing the vehicle’s pace were excluded from PCA as they shift the interpretation of the shared subspace away from visuomotor coordination and were held constant in the automated group. Longitudinal car kinematics such as speed, acceleration, or braking input are informative but mainly for investigating within-group variability and are likely better suited for temporally aware dimensionality reduction methods. For the same reason, whether the low-dimensional representation is predictive of driving performance remains an open question: performance markers carry between-participant variance only under manual control, so testing this would require a within-group design with a clear operationalization of performance rather than the between-condition comparison pursued here. Third, although the study focused on understanding the dimensionality of driving behavior around critical events, additional low-dimensional timepoints also arise outside critical events. Visual inspection suggested that these were mostly driven by external scene constraints, such as driving through a tunnel. This directly relates to the interpretation of effective dimensionality. Since effective dimensionality was computed from the eigenvalue spectrum, it quantifies the shared variance among variables rather than the intrinsic importance of an event. In a tunnel segment, for instance, the environment constrains possible movements and, consequently, behavior is stereotyped (e.g., looking straight ahead, uniform steering). Thus, low effective dimensionality should be interpreted as an indicator of strong coupling between effectors given the current environmental demands, rather than as a direct marker of danger or difficulty. Fully addressing the relationship between effective dimensionality and task demands will require modeling the environment and task structure more explicitly, which is beyond the scope of the present study.

A further limitation concerns the simulated nature of the driving task. Although the immersive virtual-reality setup and physical vehicle mockup were designed to elicit naturalistic behavior, real-world driving entails additional challenges. Thus, simulator-induced discrepancies may have influenced how drivers anticipated and responded to events, potentially inflating the regularity of the low-dimensional coordination patterns relative to real-world driving. The absence of genuine physical risk and true vestibular and proprioceptive feedback may likewise have attenuated hazard responses. These factors should be considered when generalizing our findings to real-world scenarios. Finally, the incomplete demographic data restricted our ability to rule out residual imbalances between groups or to examine how variables like age, sex, or driving experience might influence the low-dimensional behavior structure. However, random assignment of participants to driving conditions ensured group comparability against selection biases. In particular, without information on whether participants held a driver’s license, our results cannot be assumed to reflect experienced drivers. Two aspects of the study mitigate this concern. First, before the main drive, all participants completed a standardized, condition-specific training scene with two practice events, which familiarized them with the vehicle, the virtual environment, and the task. Second, the study was conducted in Germany, where mobility and traffic education is part of the school curriculum and conveys traffic rules and hazard anticipation from an early age [74]; participants can therefore be expected to be familiar with traffic situations in general, though not necessarily with operating a vehicle. Moreover, our aim was to characterize the structure of complex natural visuomotor coordination rather than driving competence. Nonetheless, the extent to which the observed patterns generalize to expert drivers remains to be established. Future research could address these limitations to investigate whether and how these factors modulate the observed coordination patterns.

Despite these limitations, this study demonstrates that complex, natural visuomotor coordination is confined to a low-dimensional space whose effective dimensionality dynamically decreases around moments of heightened demand. It shows that effective dimensionality provides a compact, interpretable metric of how many dimensions behavior effectively occupies and how tightly effectors are coupled over time. Because this metric decreases both for hazard-related coordination and for more mundane episodes of environmental constraint, it is best read as an index of coupling given current demands rather than as a marker of danger or difficulty. Moreover, this study establishes that applying PCA across individuals yields a common subspace in which different populations can be projected on the same axes and directly compared. In the present case, it shows that delegating control does not dissolve the coordination structure but re-composes it, which suggests that low-dimensional behavioral dynamics could serve as a bridge between human-centered motor control theories and the design of automated driving systems. Consistent with the view that cognition is inherently situated and temporal [75], our results highlight the importance of modeling not only what individuals do, but also how the structure of their behavior evolves in a task-dependent manner as the environment changes.

## Conclusion

In summary, this study demonstrates that, despite their inherent complexity and variability, natural behaviors such as driving can be captured in a compact, low-dimensional behavioral space. Across nearly three hundred participants and ten hazardous events, behavior occupied a shared, low-dimensional subspace, and its effective dimensionality decreased systematically around hazards. The composition of that subspace changed while its size remained small, and it retained enough information to separate driving modes. Dimensionality is therefore better understood as a regulated property of natural behavior than as a fixed descriptive statistic: behavior compresses with task demands, and what it compresses onto reflects the control problem an agent currently faces. The transient reductions in effective dimensionality reflect moments in which perception and action are tightly coupled, with task-relevant coordination patterns dominating idiosyncratic variability. Manual and automated driving behavior occupied a common manifold yet traced distinct trajectories within it, suggesting that the delegation of control systematically reshapes visuomotor coordination while preserving overall structure. Finally, investigating the low-dimensional structure of unconstrained behavior will be crucial for comparing human and machine interaction strategies and for designing automated driving systems that are receptive to the context-dependent coordination patterns that characterize human behavior.

## Materials and methods

### Ethics statement

The study was conducted in accordance with the Declaration of Helsinki and approved by the Institutional Ethics Committee of the University of Osnabrück (approval number: Ethik37–2021), and written informed consent was obtained from all participants involved in this study before participation. Additionally, the photographer and individuals involved in recreating the scene in Fig 1A have given written informed consent to publish this image.

### Participants

This study involved 2,840 visitors during a year-long exhibition at the Deutsches Museum in Bonn (Germany). Participants were free to withdraw at any moment without explaining their reasons, and the experiment was terminated if they showed or expressed signs of motion sickness. As a result, many datasets were incomplete and excluded from the analysis. For this reason, the present analysis included 284 participants who completed the full driving task under a randomly assigned manual (*N* = 131) or automated (*N* = 153) condition and had valid eye-tracking data. Eye-tracking data was considered valid when participants’ gaze was successfully calibrated and validated before the drive, with a validation error below $1.5^{\circ }$ of accuracy, and when eye samples remained continuous throughout the driving task without tracking losses (recorded as zeros) indicating that the eye tracker had lost the eyes. Demographic information was available for only 168 of the participants who completed the follow-up questionnaire, including 125 males, 37 females, and 6 intersex individuals. The mean age of this group was 23.4 years (*SD* = 15.6). No analysis based on demographics was performed in this study.

### Experimental setup and procedure

In this study, we analyzed eye-tracking and behavioral data collected during a simulated virtual drive. To provide a realistic experience, the experiment integrated a head-mounted display (HMD) for stimulus presentation and a physical vehicle mockup. The data was collected using an HTC Vive Pro Eye with integrated Tobii eye tracking, 110° field of view, 1440 × 1600 pixel resolution per eye, and 0.5–1.1° accuracy (https://developer.vive.com/resources/hardware-guides/vive-pro-eye-specs-user-guide/) using the SRanipal SDK v1.1.0.1 (https://developer.vive.com/resources/vive-sense/eye-and-facial-tracking-sdk). The VR scene maintained an average frame rate of 88 FPS and was created in Unity 3D (version 2019.3.0f3, 64 bit; https://unity.com/) and was adapted from a publicly available VR toolkit created to study driving behavior [76]. The virtual driving environment recreated a real-life driving experience, covering approximately 11 km^2^ and featuring multiple animated pedestrians, vehicles, and animals, as well as ten critical hazardous situations (for details, see [77]).

The vehicle mockup comprised two adjacent stations, each equipped with a real car seat, a Fanatec CSL Elite Racing Wheel (PS4; adjustable turning angle 90–1080°, 970 g metal rim, 300 mm diameter) and CSL Elite Pedals (12-bit potentiometers; https://fanatec.com/eu-en/csl-elite-pedals?number=CSL_EP) for steering and braking, and an interactive monitor for headset operation and questionnaire input. Since the study took place in a public exhibition space, the experimental procedure was standardized across participants: the HMD and its in-headset headphones isolated participants from the external visual and auditory surroundings, and physical noise was minimized by playing a driving sound through the HMD’s headphones. A trained experimenter was present throughout each session to set up the equipment, deliver the briefing and training, monitor the drive, and debrief participants. The two stations allowed two participants to be tested simultaneously and independently under a randomly assigned manual or automated condition. In both conditions, participants remained seated behind the steering wheel to ensure a consistent field of view, facilitating a valid comparison of visuomotor coordination strategies. Regardless of the physical station, the VR consistently rendered a left-hand driving scene with the steering wheel on the left.

Before the main drive, each participant completed a standardized preparation. First, the seat position and steering wheel were adjusted for the participant. Second, the eye tracker was calibrated using the built-in Tobii routine and then validated in a dedicated fixation-cross scene; validation was accepted only when the validation error remained below $1.5^{\circ }$ of visual angle and the head stayed within about 2 in (≈5 cm) of the fixation target [76]. Upon successful calibration, instructions describing the driving task were then delivered within the VR environment. Finally, participants completed a condition-specific training scene, including two practice events, before transitioning to the main experiment [77]. During the entire experiment, participants were monitored for signs of motion sickness.

### Research paradigm

To understand the low-dimensional dynamics underlying complex natural behavior, we conducted a driving simulation using virtual reality (VR) (Fig 1A). During the drive, participants encountered ten critical hazardous events involving a sudden appearance of predefined driving hazards, such as a reindeer dashing through the street (Fig 1B and S1 Video). The onset of each event was operationalized as the moment the vehicle reached a fixed spatial trigger (an invisible collider defining the entrance to the “event zone”) that set the corresponding hazard in motion. The (static) hazard was already present and potentially visible before onset, depending on road geometry. As in other real-world critical driving scenarios, the simulation included hazards such as animals, pedestrians, vehicles, and landslides that posed driving challenges. The experiment consisted of two conditions: manual or automated driving (Fig 1C). While the automated condition required participants to sit and experience a fully automated drive (hands-off wheel), the manual drive gave participants complete control of the car’s steering wheel and pedals, and they were instructed to perform the drive as they would in real life (hands-on wheel). In both conditions, participants were seated behind the wheel. By replicating real-world driving scenarios using VR, we could safely collect and compare the dynamics of complex driving behavior across different conditions.

We collected various behavioral metrics during the driving simulation to analyze participants’ behavior and compare visuomotor coordination strategies under manual versus automated driving. Specifically, we recorded, in Unity’s 3D coordinate system (Fig 9), the positions (location in space) and orientations (the heading or rotation) of the eye, head, and car. Additionally, the car input data included steering, acceleration, and braking movements. A complete overview of all recorded variables and their definitions is provided in the project’s repository. These metrics characterize the perceptual and action schemas employed during driving and enabled us to examine the dimensionality of complex human behavior.

**Fig 9:**
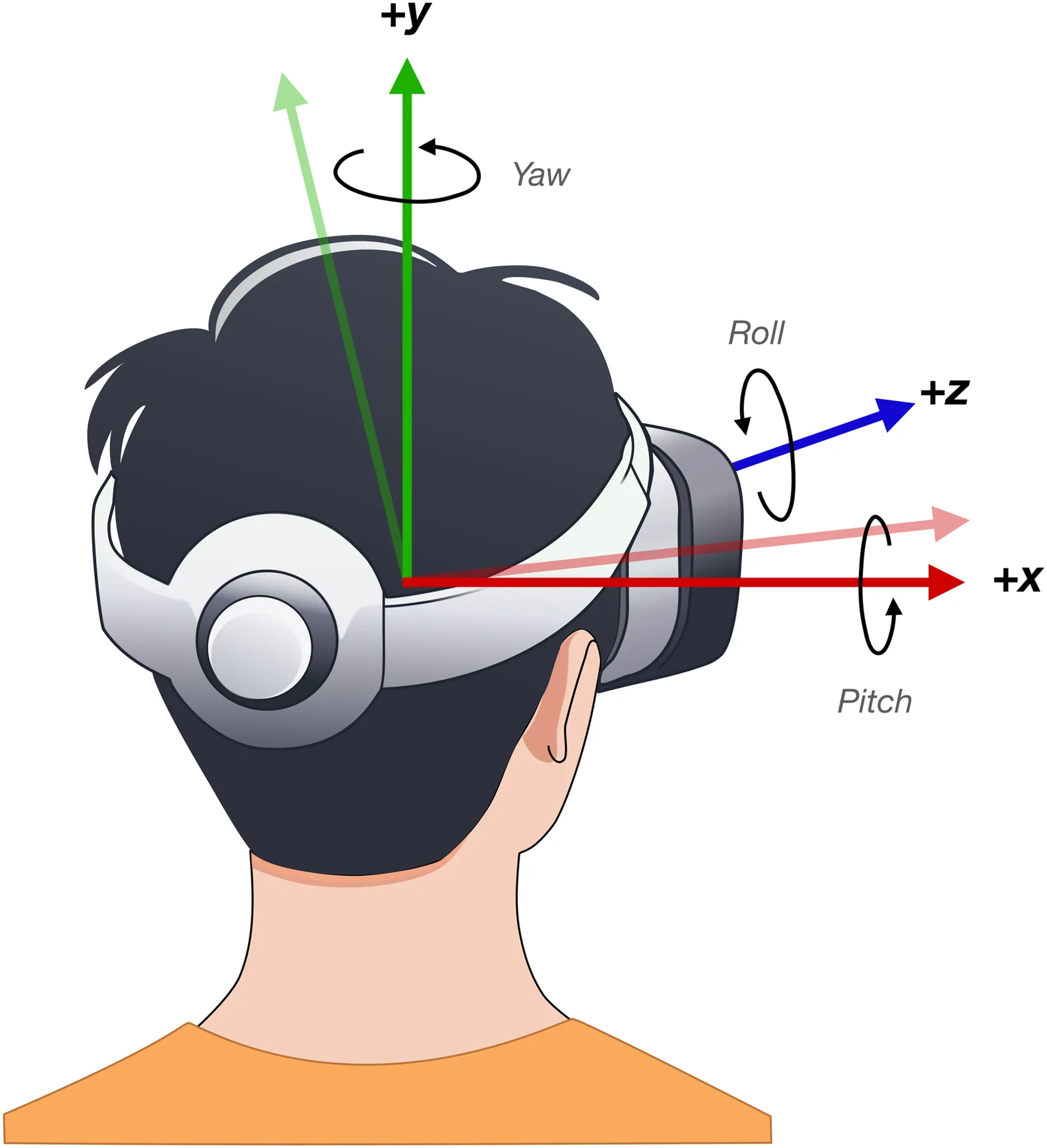
Unity 3D left-handed coordinate system representing rotations and orientations in 3D space. The z-axis points forward (blue), the x-axis points towards the right (red), and the y-axis points upwards (green). The dimmed red and green arrows depict Unity’s counterclockwise direction of rotation along +z from +x to +y. Circular arrows illustrate the Euler angles for head movements: roll (side-to-side tilt), pitch (up-and-down nod), and yaw (left-to-right turn).

### Data preprocessing

All metrics were recorded from the Unity 3D environment. Eye gaze was recorded as 3D unit vectors (position and direction) in the head reference frame, head and car orientation as quaternions in the global frame [78]; steering input was recorded in radians from the Fanatec steering wheel device and converted to degrees (Fig 9). Head orientation relative to the car was obtained by transforming global head quaternions relative to the car (parent) frame. All recordings were aligned to a standard reference time based on the car’s position from a predetermined automated drive and resampled to 50 Hz, so that events were temporally matched across participants and had comparable durations (i.e., same number of timepoints). Signals were then converted into degrees using Euler angles (see below) and screened for extreme outliers using a robust variant of the Mahalanobis distance based on the minimum covariance determinant (MCD) [79,80]. Unlike the standard Mahalanobis distance, which relies on the sample mean and covariance matrix of the full sample, the MCD estimates location and scatter from the least contaminated subset of observations. Because eye, head, and car movements differ in scale and type, the procedure was applied separately to each movement category (horizontal and vertical gaze, head pitch, yaw, and roll, car orientation, and steering input). Flagged observations were then linearly interpolated.

#### Gaze data.

Following Unity’s left-handed coordinate system and *z*-*x*-*y* rotation order [81,82], horizontal and vertical gaze angles (in degrees) were computed from the gaze-direction unit vector (*x*, *y*, *z*) using Eq. (1) and (2), respectively. Here, *x* is the lateral component, *y* the vertical component, and *z* the depth component of the unit vector.


$$\begin{array}{c} \theta_{h}=\frac{180}{\pi }\arctan\left(\frac{x}{z}\right) \end{array}$$ (1)

$$\begin{array}{c} \theta_{v}=\frac{180}{\pi }\arctan\left(\frac{y}{z}\right) \end{array}$$ (2)

#### Head data.

Head orientation was obtained from the Head Mounted Display (HMD). We converted the head quaternions to Euler angles using the Rotation class from the scipy.spatial.transform package. This process involved two steps: we first transformed global head into local, then converted it to Euler angles. With the global head quaternion $q_{head}^{global}=[x_{g},y_{g},z_{g},w_{g}]$ and the parent quaternion $q_{car}=[x_{p},y_{p},z_{p},w_{p}]$, the inverse of the unit parent quaternion is given by Eq. (3). The local head quaternion $q_{head}^{local}$ was then obtained using Eq. (4) as the product of the global head quaternion and the parent inverse.


$$\begin{array}{c} q_{car}^{-1}=\left[-x_{p},\;-y_{p},\;-z_{p},\;w_{p}\right] \end{array}$$ (3)

$$\begin{array}{c} q_{head}^{local}=q_{head}^{global}\cdot q_{car}^{-1} \end{array}$$ (4)

We then converted the local head quaternions to Euler angles following Unity’s *z*-*x*-*y* order using the as_euler property of the Rotation class Eq. (5), yielding roll (ϕ), pitch (θ), and yaw (ψ) along the z-, x-, and y-axes, respectively Eqs. (6)–(8), and then converted to degrees Eq. (9).


$$\begin{array}{c} (\phi ,\theta ,\psi )=as\_euler\;\left(q_{l},zxy\right) \end{array}$$ (5)

$$\begin{array}{c} \phi =atan2\;\left(2(xy+wz),\;w^{2}+x^{2}-y^{2}-z^{2}\right) \end{array}$$ (6)

$$\begin{array}{c} \theta =\arcsin\;\left(-2(xz-wy)\right) \end{array}$$ (7)

$$\begin{array}{c} \psi =atan2\;\left(2(yz+wx),\;w^{2}-x^{2}-y^{2}+z^{2}\right) \end{array}$$ (8)

$$\begin{array}{c} \alpha^{\circ }=\alpha \cdot \frac{180}{\pi } \end{array}$$ (9)

#### Car data.

Car rotation quaternions were converted to Euler angles in degrees using Eqs. (5) and (9). And steering input was converted from radians to degrees using Eq. (9).

### Segmentation and principal components analysis

We investigated the low-dimensional behavioral dynamics underlying driving using a structured methodology for data segmentation and dimensionality reduction via principal component analysis (PCA) [33–35]. The analysis was conducted in Python, and the results were compared with those from other statistical software [83]. Initially, the dataset comprising several behavioral variables, including eye, head, car, and steering behavior over time, was organized into a three-dimensional block structure, where each dimension represented a behavioral variable at a timepoint for each participant (Fig 2A). From this structure, we segmented the dataset into time segments spanning the complete duration of the ride, where each time segment corresponds to a single timepoint (Fig 2B). Each time segment therefore contained a single value for each variable for each participant, forming a subject × features matrix. For each time segment, PCA was applied to transform the high-dimensional parameter space (i.e., the multidimensional set of features characterizing the dataset) into a simplified and interpretable representation (Fig 2C). Before PCA, we standardized the data to have all variables in a similar unit range, treating each variable equally during analysis regardless of its original scale [35,84]. We included in the PCA the set of signals that directly index eye–head–hand–vehicle angular coordination, which define the perception–action loop that is the focus of this study. Longitudinal vehicle kinematics (e.g., acceleration, braking) were excluded from PCA because, unlike steering that indexes the hand’s angular contribution, these metrics do not share the same unit range and would shift interpretation away from visuomotor coordination.

Because PCA was applied independently at each timepoint, the procedure yielded a continuous, time-resolved sequence of PCA results: at every timepoint we obtained the eigenvalues (and thus the variance explained by each PC), the participants’ scores on each PC, and the loadings relating each behavioral variable to each PC. We used these outputs to track the variance explained across PCs over the entire drive and projected participants onto PC1 and PC2 to represent behavior within a two-dimensional subspace. Because a PCA is obtained at every timepoint, examining all timepoints individually to obtain insights into visuomotor strategies via biplots was impractical, and we therefore focused on strategic timepoints: the event onset and offset, together with two timepoints after onset defined by the cumulative variance of the first two PCs, namely its peak and its subsequent decline. These correspond, respectively, to the states of low and high effective dimensionality examined in the exemplary critical event. At these timepoints, we also quantified each variable’s association with the components as Pearson correlations between the variable and the component scores [83,85], obtaining two-sided *p*-values from the corresponding *t*-statistic. Two complementary measures derived from the PCA results are described in the following subsection.

### Cosine similarity and effective dimensionality

At each timepoint, PCA yielded two complementary descriptors of the low-dimensional representation: the squared cosine ($\cos^{2}$), which measures how well each individual variable is represented by a given principal component (PC), and the effective dimensionality, which summarizes the entire eigenvalue spectrum into the number of dimensions the representation effectively occupies. The quality of representation of each variable within each PC was assessed using the squared cosine ($\cos_{jk}^{2}$) [33,35,83], defined in Eq. (10), which quantifies the squared correlation between a variable and a PC.


$$\begin{array}{c} \cos_{jk}^{2}=\frac{{loading}_{jk}^{2}\lambda_{k}}{\sigma_{j}^{2}} \end{array}$$ (10)

where ${loading}_{jk}$ is the loading of variable *j* on component *k*, $\lambda_{k}$ is the eigenvalue of component *k*, and $\sigma_{j}$ is the standard deviation of variable *j*.

Having quantified how individual variables load onto the components, we next summarized the overall effective dimensionality (ED) of the representation. We obtained ED from the eigenvalue spectrum at each timepoint using entropy and variance-based estimators [36]. In our analysis, we employed all four estimators: *n*_1_, *n*_2_, $n_{C}$, and $n_{\infty }$. Each captures different characteristics of the eigenspectrum. *n*_1_ provides a balanced assessment, considering the distribution of variance relatively evenly across dimensions. *n*_2_ gives more weight to the dominant eigenvalues, emphasizing the contribution of dimensions with the highest variance while discounting smaller ones and serving as a reasonable lower bound. $n_{C}$ is a variance-based estimator that quantifies the dispersion of the eigenvalues, defining dimensionality as the total number of variables minus a penalty term that increases as the distribution of variance becomes more unequal. Finally, $n_{\infty }$ denotes the limiting case in which only the largest eigenvalue is considered, providing insight into dimensionality dominated by a single factor. ED thus provides an interpretable measure of the number of independent, orthogonal dimensions required to account for the overall covariation pattern observed in the data; a lower ED indicates tighter cross-variable coordination.

### Statistical analysis

To investigate event-evoked reorganization of the low-dimensional behavioral space, we analyzed changes in principal component (PC) variance and effective dimensionality (ED) relative to the onset of critical driving hazards. Conceptually, hazard onset marks a natural boundary between baseline driving and the event-related driver engagement with the hazard. Comparing a before onset baseline window with an after onset engagement window thus provides the minimal, most direct test for event-related variance reorganization. We defined symmetric before and after onset windows of ±5 s around hazard onset (excluding *t* = 0), because using a 5 s post-onset interval keeps *t*he analysis within the active phase of engagement with the hazard for even the shortest event (~7 s). The matching 5 s pre-onset window ensures baseline durations are consistent.

For each event (*N* = 10), explained variance for each PC was aligned to event onset. We evaluated whether PC variance shifted systematically between the before and after onset windows across all event × PC pairs. Because variance estimates across different events and PCs exhibit heterogeneous levels of measurement precision, characterized by varying standard errors, we applied an inverse-variance-weighted chi-square test (null hypothesis: before = after variance) [86]. This weights the variance difference of each pair by the inverse of its own variance, ensuring that highly precise estimates contribute more to the aggregate test statistic, while noisier estimates are appropriately down-weighted. In brief, we expressed each pair’s change relative to its own standard error (a standardized change) and summed the squared standardized changes into a single $\chi^{2}$ statistic; this gives more influence to those event × PC pair differences with low standard error and less to uncertain ones. We hypothesized that leading dimensions (PC1-PC2) would account for a greater share of variance after onset, whereas variance in later dimensions would decrease, indicating a reorganization of the underlying dynamics. An analogous analysis was performed to investigate differences in effective dimensionality (ED) between the before and after onset windows (null hypothesis: before = after dimensionality). For both analyses, since a $\chi^{2}$ statistic only indicates whether a difference exists, we also report the inverse-variance-weighted mean change ($\hat{\Delta }$) and its 95% confidence interval (CI) to quantify the magnitude of the shift. Together, these analyses quantify event-evoked shifts in variance and dimensionality, and assess the consistency of these dynamical transitions across diverse events.

### Behavioral subspace stability across events

To assess whether the between-subject similarity structure observed in the minimal PC1–PC2 subspace generalizes across critical events, we performed a second-order representational similarity analysis (RSA) [87]. Because PCA is calculated independently at each timepoint, the eigenvector sign, axis order, and rotation of the resulting plane are arbitrary, rendering raw subject coordinates non-comparable across events. We therefore summarized each event by a basis-free object: a subject × subject representational dissimilarity matrix (RDM) of pairwise Euclidean distances in the PC1–PC2 plane, which is invariant to any orthogonal transformation. For event *e* at timepoint *t*, the RDM entry for subjects *i* and *j* is


$$\begin{array}{c} D_{t}^{e}[i,j]=\|s_{i}^{e}(t)-s_{j}^{e}(t){\|}_{2} \end{array}$$ (11)

where $s_{i}^{e}(t)$ denotes the (PC1, PC2) coordinate of subject *i* in event *e* at timepoint *t*. To reduce noise, per-timepoint RDMs were averaged over a ±0.5 s window centered on each even*t*’s low effective dimensionality timepoint $t_{e}^{*}$:


$$\begin{array}{c} {\bar{D}}^{e}=\frac{1}{\left|W\right|}\sum_{t\in W}D_{t}^{e} \end{array}$$ (12)

yielding one window-averaged RDM per event (S1 Fig). Analysis was restricted to the 283 identity-matched participants who completed all 10 events (one participant lacked data for one event), ensuring that the RDMs index the same subjects across events. We then quantified the similarity between events’ RDMs using second-order Spearman correlations over the upper-triangle entries of each pair [86]:


$$\begin{array}{c} \rho_{ef}=Spearman(vech\;{\bar{D}}^{e},\;vech\;{\bar{D}}^{f}) \end{array}$$ (13)

producing a 10×10 second-order correlation matrix. The overall stability of the similarity structure was summarized as the mean off-diagonal correlation:


$$\begin{array}{c} S=\frac{1}{45}\sum_{e<f}\rho_{ef} \end{array}$$ (14)

To visualize the event-level structure, the 10×10 second-order correlation matrix was embedded in two dimensions via multidimensional scaling (MDS) on the dissimilarity $1-\rho_{ef}$ [87], so that inter-event distances approximate representational dissimilarities. Statistical significance of each pairwise correlation was evaluated using subject-level Mantel permutation tests [86,88] (1,000 permutations per event pair). That is, subject labels of one RDM were permuted and the correlation recomputed, so that the *p*-value corresponds to the fraction of permutations reaching the observed $\rho_{ef}$. False discovery rate (FDR) was controlled across the 45 event pairs using Benjamini–Hochberg (α=0.05) [89]. A 95% confidence interval for *S* was obtained via a subject bootstrap (1,000 resamples with replacement), rebuilding all RDMs on each resample to respect the dependence structure among subject pairs. The primary analysis was conducted on the pooled population sample (i.e., manual and automated groups combined) to assess overall cross-event group stability, and separately within each driving condition to compare the stability of the similarity structure between groups (S1 Fig).

### Discriminant analysis and cross-validated AUC

After applying PCA and reducing the behavioral variables to the first two principal components, we tested whether this compact, low-dimensional representation of multi-signal behavior captures reliable condition separation, both geometrically and predictively. To ensure comparability of axes across time, the 2D loadings matrix was aligned to a reference basis using orthogonal Procrustes rotation with reflection suppression, followed by component-wise sign continuity [34]. Condition separation was quantified directly in this aligned PC plane by computing the class centroid for automated (label 0) and manual (label 1), forming the unit discriminant axis along the centroid difference, and projecting individual PC scores onto this axis. Group-mean projections with bootstrap 95% confidence interval (CI) were obtained to report the discriminant score. The Euclidean distance between class centroids yields the reported centroid-distance (with bootstrap CI). For classification performance, we trained a logistic regression classifier at each timepoint using stratified 5-fold cross-validation [90,91]. We report the area under the receiver operating characteristic curve (AUC-ROC) scores from the out-of-fold probabilities as a measure of the discriminatory power of the model (AUC ≤ 0.5 indicates random chance; AUC = 1 indicates perfect discrimination). Statistical inference used subject-level label permutation to preserve within-subject temporal dependence: we repeatedly permuted the subject-to-label assignment and, at each timepoint, recomputed AUC and centroid distance to obtain permutation distributions. Two-sided *p*-values were estimated, and false discovery rate (FDR) was controlled using Benjamini–Hochberg (α=0.05) applied independently across time for each metric.

## Supporting information

S1 Video Exemplary driving events. A video showing exemplars of various critical events, including the training scene, a lane closure, a roadway obstruction, and a pedestrian intrusion.
(MP4)

S2 Video Four-panel composite of a driving hazard. Synchronized view of a single driving event. (Top left) exemplar video of the drive. (Top right) cosine scores. (Bottom left) explained variance of each principal component over time (seconds). (Bottom right) PCA biplot. All panels are aligned to a common time axis. A slow-motion effect was applied within the hazard-related time window. The composite illustrates how temporal changes in variance and loadings relate to the driving behavior.
(MP4)

S3 Video Biplots and cosine matrix for a driving event. A video showing: (left) the projections of the behavioral variables onto the first two principal components (PC1 and PC2) over time for an exemplary event; (right) the cosine similarity of the variables with the components.
(MP4)

S1 Table Variables’ correlations with PC1 and PC2. A table containing the correlations of the variables with the first two principal components at different timepoints of the drive during event one; *p*-values, ranks, and cumulative variance are also provided.
(PDF)

S1 FigWindow-averaged between-subject representational dissimilarity matrix and its within-window variability for a representative event.Underlying representational structure for a single critical event (event one) in the minimal PC1–PC2 subspace, computed over the ±0.5 s window centered on that event’s low effective dimensionality anchor $t_{e}^{*}$. (A) Window-mean representational dissimilarity matrix (RDM) grouped by condition (Manual, blue; Automated, red). Each cell in the matrix is the mean pairwise Euclidean distance between two subjects’ coordinates (PC1, PC2) averaged across the window’s timepoints (darker = more similar). (B) Density distribution of the window-mean pairwise distances for within-condition pairs. (C) Within-window variability of each pairwise distance, quantified as the coefficient of variation (CV = SD/mean) across the window’s timepoints; low CV indicates that a pair’s distance is stable over the averaging window. (D) Density distribution of the within-window CV for within-condition pairs. The low and narrowly distributed CV values (panels C and D) confirm that the per-timepoint RDMs vary little within the window, justifying the window-mean approach used to define each event’s RDM in the second-order representational similarity analysis.(PDF)
