# Supplementary material for: Visuomotor coordination on the road: Low-dimensional representations reveal adaptive, context-dependent reductions in the dimensionality of natural driving behavior
Source: PLoS Comput Biol. 2026 Oct 23;22(10):e1014056. doi: 10.1371/journal.pcbi.1014056 (PMC13722334; doi:10.1371/journal.pcbi.1014056)
Supplement: Supplementary file 1 [file pcbi.1014056.s004.pdf]

**S1 Table. Variables' correlations with PC1 and PC2.** A table containing the correlations of the variables with the first two principal components at different timepoints of the drive during event one; $p$-values, ranks, and cumulative variance are also provided.

| Times | PC | Variable | $r$ | $p$-value** | Rank | $n$ | Variance |
|---|---|---|---|---|---|---|---|
| Onset 20.84 s | PC1 | Head Roll | 0.8428 | $7.56 \times 10^{-78}$ | 1 | 284 | 0.2671 |
| | | Eye Horizontal | 0.1910 | $1.22 \times 10^{-3}$ | 2 | | |
| | | Head Yaw | -0.2031 | $5.75 \times 10^{-4}$ | 3 | | |
| | | Head Pitch | -0.6694 | $2.86 \times 10^{-38}$ | 4 | | |
| | | Eye Vertical | -0.7987 | $3.60 \times 10^{-64}$ | 5 | | |
| | PC2 | Eye Horizontal | 0.6393 | $4.99 \times 10^{-34}$ | 1 | 284 | 0.1922 |
| | | Head Pitch | 0.3271 | $1.66 \times 10^{-8}$ | 2 | | |
| | | Car Yaw | 0.1273 | $3.20 \times 10^{-2}$ | 3 | | |
| | | Steering | -0.6043 | $1.18 \times 10^{-29}$ | 4 | | |
| | | Head Yaw | -0.6700 | $2.33 \times 10^{-38}$ | 5 | | |
| Low ED* 23.74 s | PC1 | Head Roll | 0.8524 | $2.23 \times 10^{-81}$ | 1 | 284 | 0.3395 |
| | | Steering | 0.2745 | $2.66 \times 10^{-6}$ | 2 | | |
| | | Head Yaw | -0.1546 | $9.09 \times 10^{-3}$ | 3 | | |
| | | Head Pitch | -0.8220 | $6.27 \times 10^{-71}$ | 4 | | |
| | | Eye Vertical | -0.9372 | $4.94 \times 10^{-131}$ | 5 | | |
| | PC2 | Steering | 0.2997 | $2.64 \times 10^{-7}$ | 1 | 284 | 0.1983 |
| | | Head Yaw | -0.3540 | $8.26 \times 10^{-10}$ | 2 | | |
| | | Eye Horizontal | -0.7412 | $9.69 \times 10^{-51}$ | 3 | | |
| | | Car Yaw | -0.7794 | $3.38 \times 10^{-59}$ | 4 | | |
| High ED* 28.66 s | PC1 | Head Pitch | 0.8577 | $1.94 \times 10^{-83}$ | 1 | 284 | 0.2683 |
| | | Eye Vertical | 0.8451 | $1.17 \times 10^{-78}$ | 2 | | |
| | | Car Yaw | -0.1292 | $2.95 \times 10^{-2}$ | 3 | | |
| | | Head Yaw | -0.1521 | $1.03 \times 10^{-2}$ | 4 | | |
| | | Head Roll | -0.6254 | $3.12 \times 10^{-32}$ | 5 | | |
| | PC2 | Eye Horizontal | 0.5623 | $4.49 \times 10^{-25}$ | 1 | 284 | 0.1828 |
| | | Car Yaw | 0.4409 | $6.12 \times 10^{-15}$ | 2 | | |
| | | Head Roll | 0.4131 | $3.96 \times 10^{-13}$ | 3 | | |
| | | Eye Vertical | 0.1440 | $1.51 \times 10^{-2}$ | 4 | | |
| | | Steering | -0.3100 | $9.61 \times 10^{-8}$ | 5 | | |
| | | Head Yaw | -0.6941 | $3.77 \times 10^{-42}$ | 6 | | |
| Offset 30.98 s | PC1 | Head Roll | 0.7659 | $5.05 \times 10^{-56}$ | 1 | 284 | 0.3054 |
| | | Eye Horizontal | -0.2780 | $1.96 \times 10^{-6}$ | 2 | | |
| | | Car Yaw | -0.2948 | $4.22 \times 10^{-7}$ | 3 | | |
| | | Head Pitch | -0.8243 | $1.27 \times 10^{-71}$ | 4 | | |
| | | Eye Vertical | -0.8370 | $8.38 \times 10^{-76}$ | 5 | | |
| | PC2 | Head Yaw | 0.6822 | $3.16 \times 10^{-40}$ | 1 | 284 | 0.1952 |
| | | Steering | 0.6027 | $1.80 \times 10^{-29}$ | 2 | | |
| | | Head Roll | -0.2769 | $2.15 \times 10^{-6}$ | 3 | | |
| | | Car Yaw | -0.6677 | $5.09 \times 10^{-38}$ | 4 | | |

* ED = effective dimensionality; ** $p$-values $< 0.05$
