# Supplementary material for: Visuomotor coordination on the road: Low-dimensional representations reveal adaptive, context-dependent reductions in the dimensionality of natural driving behavior
Source: PLoS Comput Biol. 2026 Oct 23;22(10):e1014056. doi: 10.1371/journal.pcbi.1014056 (PMC13722334; doi:10.1371/journal.pcbi.1014056)
Supplement: Supplementary file 2 [file pcbi.1014056.s005.pdf]

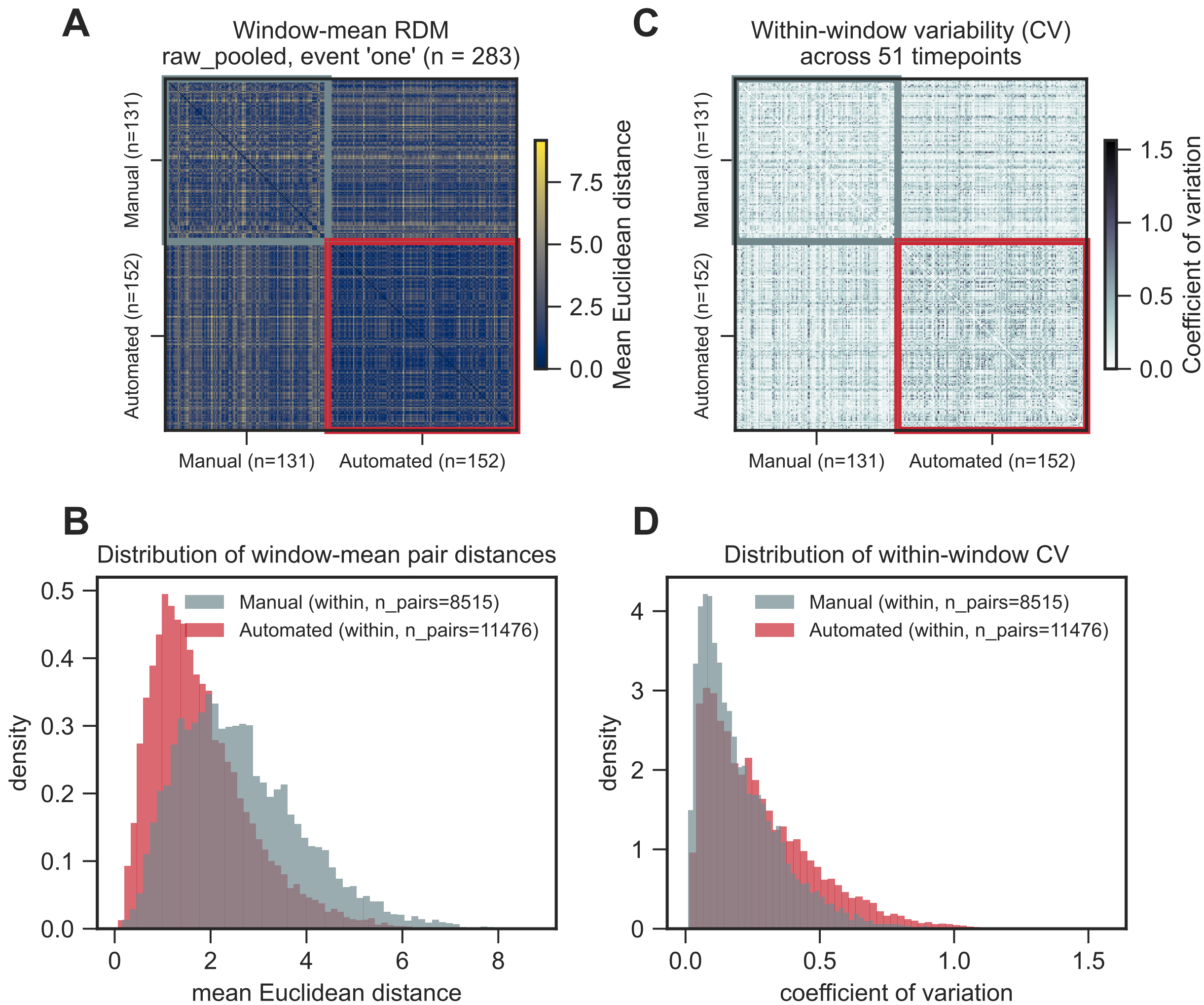

S1 Fig. Window-averaged between-subject representational dissimilarity matrix and its within-window variability for a representative event. Underlying representational structure for a single critical event (event one) in the minimal PC1–PC2 subspace, computed over the ± 0.5 s window centered on that event's low effective dimensionality anchor t_e^*. (A) Window-mean representational dissimilarity matrix (RDM) grouped by condition (Manual, blue; Automated, red). Each cell in the matrix is the mean pairwise Euclidean distance between two subjects' coordinates (PC1, PC2) averaged across the window's timepoints (darker = more similar). (B) Density distribution of the window-mean pairwise distances for within-condition pairs. (C) Within-window variability of each pairwise distance, quantified as the coefficient of variation (CV = SD/mean) across the window's timepoints; low CV indicates that a pair's distance is stable over the averaging window. (D) Density distribution of the within-window CV for within-condition pairs. The low and narrowly distributed CV values (panels C and D) confirm that the per-timepoint RDMs vary little within the window, justifying the window-mean approach used to define each event's RDM in the second-order representational similarity analysis.
