# Supplementary material for: Visuomotor coordination on the road: Low-dimensional representations reveal adaptive, context-dependent reductions in the dimensionality of natural driving behavior
Source: PLoS Comput Biol. 2026 Oct 23;22(10):e1014056. doi: 10.1371/journal.pcbi.1014056 (PMC13722334; doi:10.1371/journal.pcbi.1014056)
Supplement: Supplementary file 3 [file pcbi.1014056.s006.pdf]

Response to reviewers' comments

To: Editor and Reviewers

Subject: Responses to Reviewers' Comments for Visuomotor coordination on the road: low-dimensional representations reveal adaptive, context-dependent reductions in the dimensionality of natural driving behavior (PCOMPBIOL-D-26-00403)

Dear Editor, dear Reviewers,

We thank the editors and both reviewers for their careful reading, their constructive criticism, and the opportunity to submit a revised version of our manuscript. The comments identified key points, and addressing them has enhanced the manuscript's arguments and clarity. Each point raised has been answered with a corresponding revision. The comments prompted new analyses, yielding results that we consider genuinely informative and that are now part of the manuscript. The most substantial improvements are the following:

New analysis 1: Following Reviewer 1's suggestion, we now examine whether the between-subject organization recovered at the timepoint of low-effective dimensionality for one critical event is stable across events. Using a second-order representational similarity analysis at each event's timepoint of low-effective dimensionality, we found that events are positively and significantly correlated, with a mean second-order correlation of S = 0.28 (95% CI [0.25, 0.33]), and that this shared organization is reliably stronger among manual than automated driving groups (S = 0.34 versus S = 0.25; difference of 0.09, p = 0.035). The between-subject organization is therefore partially stereotyped across events: a consistent component of inter-individual similarity recurs at every hazard, alongside a substantial event-specific component. This analysis is reported in a new Results subsection “Between-subject similarity in low-dimensional space is partially stable across critical events”, a new Fig 7, S1 Fig, and Methods subsection.

New analysis 2: Following Reviewer 2's question of whether two components are sufficient, we performed a component-sweep ablation, retraining the cross-validated classifier on PC1 alone, on PC1-PC2, and on PC1-PC2-PC3. Mean AUC rose from 0.61 with the first component to 0.68 with the first two, whereas adding a third component yielded only a marginal gain of 0.03. The condition-discriminative information is thus already contained in the first two components, which substantiates the two-dimensional representation used throughout the paper. The ablation is now shown in Fig 8C and described in the Results.

Beyond these analyses, the manuscript now follows the PLOS section sequence (Abstract, Introduction, Results, Discussion, Methods), which renumbered the figures: former Figs 4-7 are now Figs 3-6, former Fig 3 is now Fig 9, and Figs 1, 2, and 8 keep their numbers. The Methods now give the rationale for the experimental setup and the statistical analyses, together with the previously missing method references; the Introduction clarifies driving as a complex action through which we investigate visuomotor coordination with explicit research questions; and the Discussion adds limitations on the simulated nature of the task and on the incomplete demographic information.

Below we provide a point-by-point response to the reviewers' comments and concerns, detailing how and where each point was addressed in the revised manuscript. We are confident that these revisions thoroughly address all concerns raised and have strengthened the manuscript's arguments.

Sincerely,

John Madrid-Carvajal (on behalf of all authors)

Response to Reviewer #1

General assessment: Overall, the manuscript is well written, the data are interesting and complex (and freely available), and the analysis is an interesting approach. I have mainly some comments with respect to the analysis.

Authors' response: We thank the reviewer for the positive overall assessment and for the constructive comments, which we address point by point below.

Comment 1: Methods: The authors performed an alignment of the data to a standard time. While I understand the necessity of this step for the future analysis, could this alignment hide some of the inter-participant variability? For example, some people might go overall faster and some overall slower, and as far as I see, variables like acceleration of the car were measured but are not included in the PCA. Would adding the average car speed change something in the main results?

Authors' response:

Done/addressed– We thank the reviewer for this careful observation and for understanding the necessity of the temporal alignment. To address the two related points raised, **in the revised manuscript, we have** (i) clarified in the Methods (Segmentation and principal components analysis) why longitudinal kinematics such as car speed were excluded from the primary PCA (Lines 872–877), and (ii) expanded the Discussion limitations to note speed and other longitudinal kinematics as promising variables for within-group, temporally-aware analyses (e.g., functional PCA) in future work (Lines 649–657). We address each point in turn below for further clarification.

(1) Could the alignment hide inter-participant variability? The reviewer is correct that aligning recordings to a standard reference time (defined by the car's position along a predetermined route) normalizes differences in overall pace, so that two drivers are matched at the same spatial location. This step was essential to compare the same critical events across participants on a common time base. Importantly, this alignment operates on the temporal axis only; it does not remove the between-participant variability contained in the other behavioral variables themselves, which is precisely the inter-individual covariation that our cross-sectional PCA is designed to capture.

(2) Would adding average car speed change the main results? We deliberately restricted the primary analysis to signals that directly register eye–head–hand–vehicle angular coordination, i.e., the perception–action loop that is the explicit focus of the study. Other related kinematics (speed, acceleration, braking) describe the vehicle's pace rather than the coordination among effectors, so including them would shift the interpretation of the shared subspace away from visuomotor coordination. A second, decisive reason concerns the group comparison that is the main focus of the analysis: because the automated drive followed a predetermined trajectory, car speed was identical across the automated-condition group (near-zero between-subject variance). In a cross-sectional PCA that extracts directions of inter-individual covariation, such a variable contributes essentially no variance for the automated group and would enter the shared manual versus automated subspace asymmetrically, carrying information for the manual group only. Rather than altering the low-dimensional structure we report, this would confound the very between-condition comparison that the shared space is built to support. In short, average car speed is informative mainly as a within manual group variable, which is out of the scope of this study.

Comment 2: Methods: Out of interest, did people suffer from motion sickness in the simulator? Could this explain some types of the observed behavior?

Authors' response:

Done – We thank the reviewer for this question. We monitored participants for motion sickness. Upon any indication of sickness, the session was terminated immediately; the data were excluded from analyses. The 284 analyzed participants therefore comprise only those who completed the full drive, so motion sickness is unlikely to explain the observed behavior. Additionally, certain strategies such as short overall experiment duration (approx. 10 min) and dedicated training sessions were implemented to reduce the chances of motion sickness. **In the revised manuscript**, we have clarified this in the Methods section. **Added statement:** "[...] Finally, participants completed a condition-specific training scene, including two practice events, before transitioning to the main experiment [73]. During the entire experiment, participants were monitored for signs of motion sickness." (Line 786)

Comment 3: Results: If I understand correctly, since a large part of the analysis is focused on 1 particular event, it would be really helpful to describe the event in question a bit in detail. This will help with the interpretation and understanding of the data.

Authors' response:

Done – We thank the reviewer for this helpful suggestion. **In the revised manuscript**, we have (i) added a short statement describing the event at the start of the single-event analysis, making it concise: "We focus on a representative critical event (≈ 10s duration) that, upon event onset, depicts a left-hand curve with a hazard entering the roadway soon after (Fig 1B and S2 Video)." (Lines 210–213). Further details are visible in the video. We have also (ii) clarified that the "representative event" used in the discriminant analysis (Fig 8) is this same event, so it is consistently throughout.

Comment 4: Results: One pattern that seems quite clear in Fig 7, but can also be seen in the other traces: The reduction in the effective dimensionality often starts before 0, so it is not really a sharp drop after the event, but it seems to be a predictive response that shows a preparation to respond to a future event. One could try to test whether that pattern is consistent (so when is the onset of the drop) and whether this timing differs across events, and is related to at which point in time something is visble (e.g. the merging of a lane can be seen from further away than an animal suddenly stepping on the street).

Authors' response:

Done – We thank the reviewer for this excellent observation. The reviewer is correct that the reduction in effective dimensionality frequently begins shortly before the event onset (t = 0) rather than as an abrupt post-onset drop, and that this pattern is visible across the individual event trajectories. **To make things clearer in the revised manuscript**, we have (i) corrected the ED subsection heading and the corresponding figure caption (Fig 6 in the revised manuscript; Fig 7 in the original submission), which previously stated that ED "remains stable before onset," to instead reflect that the decline begins shortly before onset (consistent with the main text); (ii) added a definition of event onset in the Methods, clarifying that it marks a fixed spatial trigger; and (iii) added a sentence in the Discussion framing the pre-onset decline as an anticipatory, preparatory response. "Notably, this decrease often began shortly before the event onset, suggesting an anticipatory, preparatory reorganization of behavior."

This early decline is expected once the definition of event onset is made explicit. In our paradigm, the onset of each critical event was operationalized as the moment the vehicle reached a fixed spatial trigger (that is, an invisible collider indicating that the car was inside the "event zone") that set the corresponding hazard object(s) in motion. Because this trigger is defined by the vehicle's position along the route rather than by the hazard's visibility, the hazard was typically already present (static) in the scene and could be seen

before onset depending on road geometry. The onset therefore marks the start of the hazard's motion, not the moment the hazard first became visible or came into close proximity with the vehicle. Consequently, depending on the event geometry, the upcoming hazard could enter the driver's visual field earlier than t = 0.

Because this spatial trigger is identical across participants, the pre-onset window is directly comparable across drivers, so the early decline is a systematic feature of the data rather than an alignment artifact. In line with the reviewer's reading, we had described that the "sharp and rapid decline in ED began shortly before onset" (second paragraph of ED section). We interpret it as an anticipatory, preparatory reorganization of behavior: drivers begin to simplify and couple their eye–head–hand coordination as soon as the situation becomes perceptible, ahead of the triggered hazard motion. This is fully consistent with our central account of a task-relevant, low dimensional regime engaged around hazards. A formal test of whether the onset of this decline tracks each hazard's visibility would require operationalizing per-event, per-participant first-visibility (which is gaze and occlusion dependent), and we regard this as a valuable direction for dedicated future work.

Comment 5: Results: Potentially additional interesting analysis: When you have the subspace of minimal behavior for one event, you get the similarity of subjects in that space for one event. Is this scaling comparable to how similar people where in response to other events? Or in other words, is how people behave in the low-dimensional space stable?

Authors' response:

Done – We thank the reviewer for this suggestion, **which triggered new analysis, and is now added to the revised manuscript.** We have analyzed the between-subject similarity in low-dimensional space across all events and added the analysis to the revised manuscript. In this reply, we condense how we quantified this and the results obtained; the complete details are given in the manuscript. Because PCA is computed independently at each timepoint, the sign, order, and rotation of the resulting plane are arbitrary, so raw subject coordinates were not compared directly across events. We therefore summarized each event by a basis-free object, that is, a subject x subject representational dissimilarity matrix of pairwise Euclidean distances in the PC1-PC2 plane at that event's low effective dimensionality timepoint, and compared these matrices across events using second-order representational similarity analysis (new Methods subsection "Behavioral subspace stability across events" (Lines 953–986); the underlying structure for a representative event is shown in S1 Fig). **Results show that:** (i) across the 283 identity-matched participants, all 45 event pairs were positively and significantly correlated ($p < 0.001$, FDR-adjusted) (Fig 7A below) with a modest mean second-order correlation ($S = 0.28$, 95% CI [0.25, 0.33]), indicating that the arrangement of drivers in the compressed space combines a statistically reliable shared component with a substantial event-specific one. (ii) Differences between events were clearer in the two-dimensional embedding obtained via multidimensional scaling (Fig 7B). (iii) The same pattern held within each driving condition and was reliably stronger among manual than automated driving ($S = 0.34$ versus $S = 0.25$; delta-S = 0.09, $p = 0.035$), consistent with the tighter visuomotor coupling required when actively controlling the vehicle (Fig 7C-D below). **These results are reported in detail in the newly added Results subsection** "Between-subject similarity in low-dimensional space is partially stable across critical events" (Lines 417–470) with the accompanying new Fig 7 (included below). We have also carried the conclusion drawn from this stability analysis through to the Abstract, to the third research question in the Introduction, and to the Discussion.

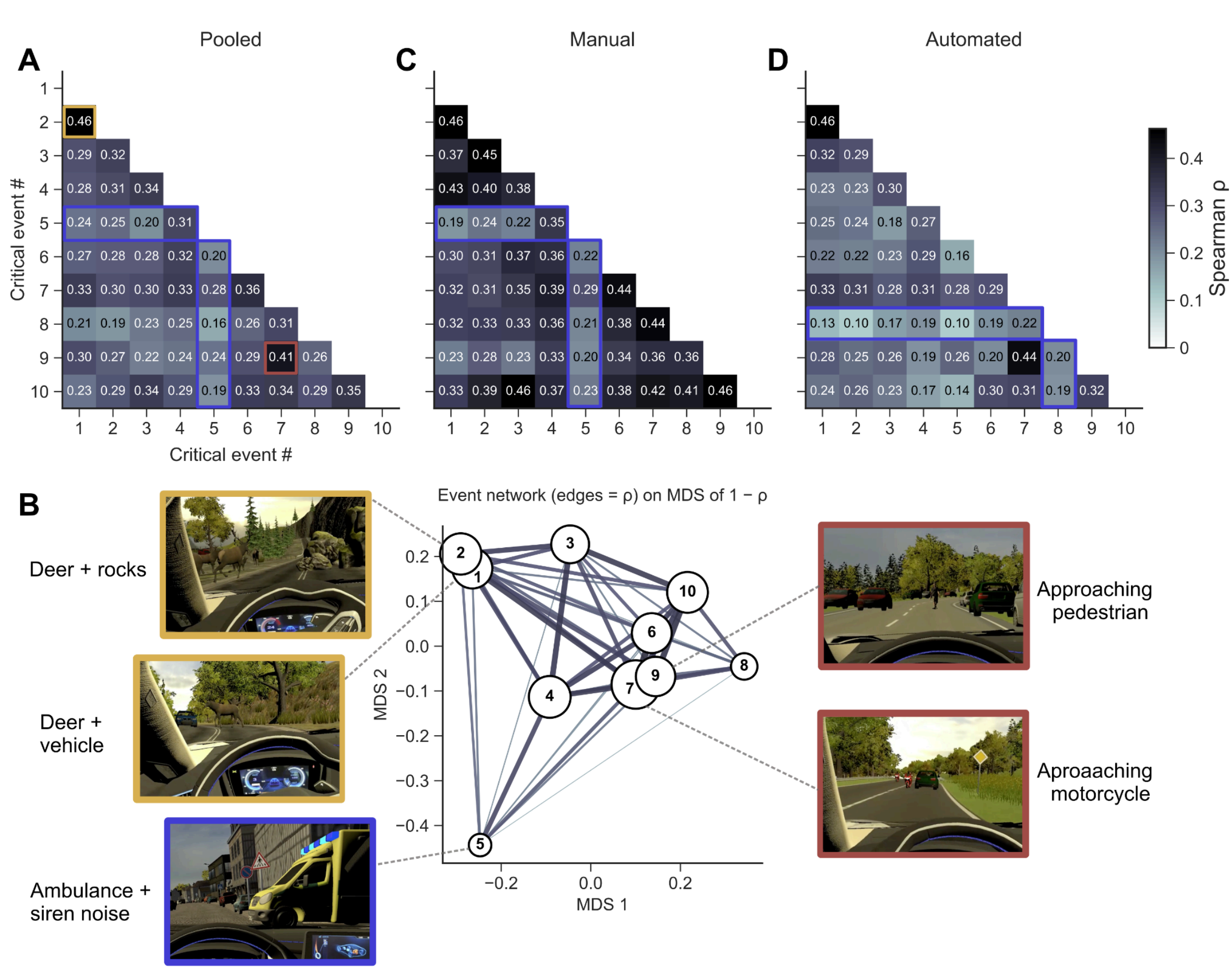

Comment 6: Results: Is there any way to link the behavior to actual performance? I guess this would be only interesting for the manual condition, but is the low-dimensional representation predictive of some objective performance markers, e.g. the smoothness of the steering, Amount of breaking or minimum distance to an obstacle?

Authors' response:

Done/addressed – We thank the reviewer for the comment. We agree that linking behavior to actual performance from a low-dimensional approach is an interesting question, requiring a within-group design with an explicit operationalization of performance. **In the revised manuscript, we have made two changes in response.** First, we updated a misleading sentence in the Methods (Research paradigm), which before used to read *"to analyze participants' responses and compare behavioral performance under manual versus autonomous driving"* and now reads: *"We collected various behavioral metrics during the driving simulation to analyze participants' behavior and compare visuomotor coordination strategies under manual versus automated driving."* The study's objective is thereby stated accurately and consistently with the aims set out in the Introduction and with the variables entering the analysis.

Second, we expanded the limitations in the Discussion (second limitation paragraph) to state that testing whether the low-dimensional representation predicts objective performance markers would require a within-group design with an explicit operationalization of performance. **Added statement:** "Longitudinal car kinematics such as speed, acceleration, or braking input are informative but mainly for investigating within-group variability and are likely better suited for temporally aware dimensionality reduction methods. For the same reason, whether the low-dimensional representation is predictive of driving performance remains an open question: performance markers carry between-participant variance only under manual control, so testing this would require a

within-group design with a clear operationalization of performance rather than the between-condition comparison pursued here.”

Comment 7: Line 117: trajectories of eye, head and car along with their positions? Isn't the trajectory made out of the different positional measurements? That sentence is a bit unclear to me.

Authors’ response:

Done – We thank the reviewer for pointing out this ambiguity. The reviewer was right; what we meant was that we recorded the positions (location in space) and orientations (the heading or rotation) of the eye, head, and car. **In the revised manuscript**, we have rewritten the sentence in the Research paradigm section and removed the word "trajectories" entirely. We also pointed to the definitions of these signals, which were also included in a table on the project’s repository (Lines 810–814).

Comment 8: Line 153: How are valid eye tracking data defined? Was there some custom calibration?

Authors’ response:

Done – We thank the reviewer for this comment. We agree that clarification on the calibration procedure and the definition of valid eye-tracking samples was missing. **In the revised manuscript**, we now define the criterion for valid eye-tracking data in the Participants section: data were considered valid when the participant's gaze was successfully calibrated and validated before the drive (validation error below 1.5°) and when eye samples remained continuous throughout the task, without tracking losses (recorded as zeros). We have additionally described the custom calibration and validation procedure in the Experimental setup section.

Comment 9: Line 163: Within a time segment, did you average the data for the individual measurements (e.g. average eye vertical position?) Please clarify.

Authors’ response:

Done – We thank the reviewer for pointing out this ambiguity. No, we did not average measurements within a time segment. Each time segment corresponds to a single timepoint (one sample), so every participant contributes a single instantaneous value per variable, which are stacked across participants into a subject × features matrix that is then submitted to PCA. This is possible because, as stated in the preprocessing section, all recordings were aligned to a common reference time and resampled to a shared 50 Hz grid, giving every participant the same number of timepoints. **In the revised manuscript**, we have clarified this in the Segmentation and principal components analysis subsection. We now state that each time segment corresponds to a single timepoint (with one value per variable per participant) and reinforced it in the Data preprocessing subsection (noting the same number of timepoints across participants).

Comment 10: Line 272ff: The abbreviations CV and FDR are introduced, but not explained. It might be helpful to do so.

Authors’ response:

Done – We thank the reviewer for pointing this out. **We have clarified these abbreviations and ensured that all abbreviations in the manuscript are defined together with their full term at first use.** The false discovery rate (FDR) is now spelled out at its first mention and used consistently thereafter. Regarding CV: in the revised manuscript, the cross-validation procedure is written out in full ("stratified 5-fold cross-validation"), so this abbreviation is no longer used in that context. The only remaining occurrence of "CV" is now in the S1 Figure and refers to the coefficient of variation, which

is defined where it appears ("coefficient of variation, CV = SD/mean"). We trust that this resolves the reviewer comment.

Comment 11: Figure 6: In the PDF version, the quality of the Figure is unfortunately not great, it is very hard to see especially the smaller labels and numbers in Panels B & C. Please make sure, that the quality is good enough. One way to improve the figure could potentially is to only highlight the first two figures in Panel B & C, since the other two are not discussed in the text. Also in Panel A, it might be helpful to align the time axis to 0 at event onset.

Authors' response:

Done – Figure quality and legibility. We thank the reviewer for the suggestion to improve the figures. **We have actually updated all images**, including this figure (Figure 5 in the revised manuscript; Figure 6 in the original submission), increasing font size and overall readability. As suggested, we have also aligned the time axis to event onset for Figures 5 and 8. Regarding the suggestion to show only the first two time points in Panels B and C: we have retained all four time points, as together they illustrate the full temporal progression from low to high effective dimensionality. Given the marked improvement in resolution and label size, all four panels are now clearly readable. We believe these changes fully address the reviewer's concerns and have improved the clarity of the figures.

Comment 12: Figure 8: Again, it could potentially be helpful to change the time axis with 0 as event onset

Authors' response:

Done – We thank the reviewer for the comment. **In the revised manuscript,** we have edited Fig 8 with the time axis aligned to 0 at event onset.

Response to Reviewer #2

General assessment: The large number or participants is very positive, in particular considering that this is a simulator study. Using and open exhibition space to collect the data is a very nice idea. Even though wth some drawbacks (see my later comments)

Overall, I like the idea of the approach and the insights that are generated even though they are mostly of theoretical interest

The writing is adequate

The figures are informative and are a valuable extension to the text. The overall visual appearance is good

I appreciate the effort that the authors put into the OSF project and overall documentation and that they share their data, code and other artifacts

Authors' response: We thank the reviewer for these encouraging overall remarks and the useful comments, which we address point by point below.

Comment 1: The structure of the introduction needs improvement. Currently, it alternates between motivation, methods, and related work, which makes it very hard to follow and confuses the reader. Furthermore, the driving use case is introduced very late. It is in the title, yet the reader has to get through several pages before it is first mentioned.

Authors' response:

Done – We thank the reviewer and agree with both points. **In the revised manuscript**, the Introduction has been substantially updated. The driving use case is now introduced in the first paragraph of the Introduction, where we state explicitly that driving serves as the paradigm to investigate complex, natural behavior. To remove the alternation between motivation, methods, and related work, we merged the paragraph describing PCA with the subsequent related-work paragraph (see also our response to the comment on the PCA description), so that the Introduction now proceeds from motivation, to the relevant concepts and prior work, to the paradigm, and finally to our research questions. We further added a brief justification of this paradigm at the end of that paragraph: driving is a complex skill acquired in adulthood and trained in a common, systematic way, which is precisely what makes coordination strategies comparable across individuals. Finally, we reformulated the closing question of the following paragraph so that it now asks a general question about complex natural behavior rather than a question about driving specifically.

Comment 2: No research questions are formulated, even though hypothesis testing is performed. Why is that? Positioning this work as a clearly explanatory study would help

Authors' response:

Done – We thank the reviewer for pointing this out. **The Introduction has been considerably updated** and now formulates the explicit research questions about the organization of complex natural human behavior, replacing the previously implicit framing of our aims. These questions are worked through consistently in the remainder of the manuscript and addressed by our analysis.

Comment 3: The study set-up is not really clear: How controlled were the surroundings (sound, etc.) given that the study was conducted in an open exhibition space? Was an experimenter present at all times? How were participants briefed? There are a lot of open questions here. Furthermore in the picture in Fig1 the steering wheel is on the right in the physical simulator but on the left in the VR simulation. How does this go together? Aslo why are there two people with hedsets in the simulator. Was this the study set-up?

Authors' response:

Done – We thank the reviewer for these questions, which helped us realize that the study setup needed more clarification. **We have substantially expanded the "Experimental setup and procedure" section, which now addresses each point:**

• Environmental control. Although the study was run in a public exhibition space, each participant was fully immersed in VR: the head-mounted display (HMD) and its in-headset audio isolated participants from the surrounding visual and auditory environment, and physical noise was minimized by playing a driving sound through the HMD's headphones.

• Experimenter presence. A trained experimenter was present throughout every session: during equipment setup, calibration and validation, briefing and training, monitoring of the drive, and debriefing.

• Briefing. Task instructions describing the driving condition and scenario were delivered within the VR environment, and each participant completed a condition-specific training scene with two practice events before transitioning to the main drive.

• Two participants with headsets (Fig 1). The vehicle mock-up comprises two adjacent stations, allowing two participants to be tested simultaneously and independently; Fig 1 shows both stations in use, which is why two headset-wearing participants are visible.

• Steering-wheel side (Fig 1). Regardless of the physical station used, the VR consistently rendered the same left-hand-drive configuration (steering wheel on the left). The apparent left/right steering-wheel difference between the physical rig and the VR view in Fig 1 therefore reflects this standardization of the simulated scene, not a difference in the task participants performed.

These clarifications appear in the revised "Experimental setup and procedure" subsection.

Comment 4: No demographic information is provided, which makes it hard to assess the comparability of the two groups and is a serious threat to validity that should be discussed in the limitations section in the discussion. It is also not clear if participants were able to choose if they want to perform the automated or manual drive. This might introduce a bias between the groups

Authors' response:

Done – We thank the reviewer for raising these points about group comparability and potential bias given the lack of complete demographic data.

• Regarding assignment and bias: participants did not choose their condition. They were randomly assigned to the manual or automated drive. **In the revised manuscript, we have edited the Methods to make this explicit** (in "Participants" and "Experimental setup and procedure"). This randomization safeguards the two groups against systematic selection differences and addresses the concern that self-selection could bias the comparison.

• Regarding demographics: demographic information is in fact reported in the Participants subsection, but only for the 168 participants who completed the voluntary follow-up questionnaire (125 males, 37 females, 6 intersex; mean age 23.4 years). Because these data were incomplete and not the focus of our analysis, **in the revised manuscript**, first we explicitly state that no analysis based on demographics was performed (in the Methods - Participants subsection). Second, following the reviewer's suggestion, **we now explicitly acknowledge this in the Discussion limitations**, noting that incomplete demographics restrict our ability to fully rule out residual between-group imbalances or to assess how factors such as age, sex, or driving experience might modulate the low-dimensional structure, while emphasizing that future work could test whether these factors modulate the observed patterns.

Comment 5: Given the videos that were attached it becomes obvious that the physics and driving behavior of the automated car are somewhat unrealistic. Simulator-induced limitations should be mentioned in the discussion

Authors' response:

Done – We thank the reviewer for this observation. We agree that a driving simulator, although immersive, cannot fully reproduce real-world driving. **In the revised manuscript**, we have added a dedicated limitation to the Discussion acknowledging the simulated nature of the task and its consequences for our findings (starting at Line 674). Specifically, we now note that simulator-induced discrepancies may have influenced how drivers anticipated and responded to events and could inflate the regularity of the observed low-dimensional coordination patterns relative to real-world driving, and that the absence of genuine physical risk and of true vestibular and proprioceptive feedback may have attenuated hazard responses. We accordingly caution that these factors should be considered when generalizing our findings to real-world scenarios.

Comment 6: The statistical analysis needs clarification. It is very hard to follow why specific decisions were made (comparing BO vs. AO, the ED abbrevaton is not introduced on first use, why did the authors choose a 5 second window size?). The choice of statistical tests is also not properly explained, for instance, why was an inverse-variance-weighted chi-square test used? Overall, I had to re-read this section multiple times and am still unsure why the analysis is designed in exactly this way. The paper would benefit from a clearer conceptual explanation of the overarching approach

Authors' response:

Done – We thank the reviewer for pointing out that the statistical analysis was hard to follow. **We have rewritten the Statistical analysis section to make the rationale for each choice explicit.** First, the overarching approach and before vs. after. We now open the section with the conceptual logic of the analysis and state why we contrast a before-onset with an after-onset window, namely that hazard onset is the natural boundary between baseline driving and the event-evoked response, making this the most direct test of event-related reorganization. Second, for the 5 seconds (s) window, we now justify the window size. A 5 s window is the largest symmetric interval that remains within the active engagement phase of even the shortest event (~7 s), keeping the before- and after-onset windows matched. Third, regarding the inverse-variance-weighted chi-square test, we now explain why this test was used and include its reference. Across all the PC X pairs comparisons, we express each before vs. after variance and effective dimensionality (ED) change relative to its own standard error and sum the squared standardized changes into a single chi-square, which gives more weight to changes with low standard error. Because a chi-square indicates only whether a difference exists, we additionally report the inverse-variance-weighted mean change and its 95% confidence interval as an effect size. Finally, the ED abbreviation: Effective dimensionality (ED) is now defined at its first mention and used consistently thereafter.

Comment 7: "The PCs' variance distributions across all events show a clear variance redistribution" - I do not agree. PC2-PC5 are nearly indistinguishable, and the insight one can draw from this seems negligible in practice due to the extremely small effect size

Authors' response:

Done – We thank the reviewer for this observation. We agree that, in the aggregated cross-event analysis (Fig 4; Fig 5 in the original submission), the before- after onset variance differences among PC2–PC5 are small and largely overlapping, and that the pooled variance change ($\hat{\Delta} = -0.002$) indicates a modest effect size despite statistical significance. **We have revised the Results text accordingly**: we no longer describe a "clear" redistribution across all PCs, and we now state explicitly that event-evoked reallocation is driven primarily by PC1, with smaller changes in PC2 and largely indistinguishable shifts among PC2–PC5. We also updated the Fig 4 caption and the corresponding Discussion sentence to align with this interpretation.

Edited statement: "Across events, explained variance shifted modestly after onset (Fig 4). This redistribution was driven primarily by PC1, which captured a larger proportion of variance after onset (mean shifts upwards), indicating event-evoked reorganization. The remaining PCs captured less variance; PC2 through PC5's variance remained stable between before and after onset windows, and PC6 and PC7's variance decreased after onset (mean shifts downwards)."

Comment 8: The connection between the many analyses performed and the actual, concrete learnings about driver behavior is often unclear. In the discussion, the authors state that "Together, these findings indicate that the visuomotor coordination underlying driving behavior is both low-dimensional and context-sensitive, with hazard-related compression revealing group-specific coordination patterns." A provocative but fair question here is: "So what?" Stronger contextualization within the driving literature - and a clearer account of how these results could improve our understanding of how people drive, or even inform better automated systems - would give the paper considerably more leverage

Authors' response:

Done – We thank the reviewer for this fair and useful challenge. **We have revised the Discussion so that it states what is actually learned rather than only what was found.** The sentence quoted by the reviewer has been rewritten: we now conclude that complex natural behavior is not merely low-dimensional, but that its dimensionality is actively regulated, with the number of effective dimensions and the variables constituting them changing independently. We further added an explicit account of how the analyses build on one another, so that the progression from establishing a low-dimensional description, to testing whether its dimensionality varies with context, to examining what the leading dimensions are composed of, to asking whether between-subject similarity recurs across events and whether the subspace still distinguishes driving conditions, is now traceable as a single line of reasoning.

Two concrete consequences are now stated explicitly. First, because the compression begins before the hazard has fully unfolded, effective dimensionality indexes anticipatory rather than reactive control, so the timing of compression offers a candidate readout of when a driver commits to a course of action. Second, because the number of dimensions remained small while their composition changed, a low-dimensional description does not discard behavioral detail but changes the basis in which it is expressed, and it is this composition, rather than the count, that distinguishes a driver controlling the vehicle from one monitoring an automated system.

Regarding contextualization, **in the revised manuscript,** we have clarified throughout the Introduction that driving serves as our research paradigm and as an example of complex natural human behavior. Our aim is to investigate visuomotor coordination during such behavior, which remains understudied given that existing work concentrates on non-human species and on constrained, repetitive tasks such as locomotion and grasping. Making this scope explicit has also given the manuscript a clearer overall structure, connecting the Introduction, Results, and Discussion around the same set of questions. Within this scope, the revised Discussion states several driving-related learnings that we hope will be of direct interest to the driving research community. Gaze remains coupled to the vehicle's heading in both conditions, actively together with steering during manual driving and passively in the absence of steering control under automation, so that delegating control re-composes rather than dissolves the underlying coordination structure. In addition, effective dimensionality provides a compact and interpretable index of how tightly effectors are coupled over time, and applying PCA across individuals yields a common subspace in which different populations can be projected onto the same axes and compared directly, which we see as a bridge between human-centered motor control accounts and the design of automated driving systems. We hope these findings will inspire more targeted analyses within the driving literature that build on this framework.

Comment 9: The section structure is somewhat unusual, especially in the Materials and Methods, where the analysis is discussed before the processing, and the ethics statement appears in the middle

Authors' response:

Done – We thank the reviewer for pointing this out; **we have now reorganized the sections order.** Specifically, the Methods so that preprocessing precedes analysis, relocated the Ethics statement, and renamed the "Materials and procedure" to "Experimental setup" for clarity. The overall manuscript is now organized according to PLOS structure.

Comment 10: The very detailed description of what PCA is in the introduction seems misplaced. In particular, if one considers how many statistical concepts and methods are not introduced in the analysis part of the paper

Authors' response:

Done – We thank the reviewer for pointing this out. **We have condensed the description of PCA in the Introduction and merged it with the following paragraph into a single, streamlined paragraph.** The PCA account is now conceptual rather than technical, which we feel is more appropriate for an Introduction: it briefly states what PCA produces (principal components and their eigenvalues) and how the eigenvalue spectrum yields effective dimensionality. This revised paragraph now appears as the third paragraph of the Introduction.

Comment 11: Effective dimensionality is introduced without explaining what it is, or why knowing it is necessary or beneficial

Authors' response:

Done – We thank the reviewer for this helpful comment. **In the revised manuscript**, we have clarified both what effective dimensionality (ED) is and why quantifying it is beneficial.

In the Introduction, where ED is first introduced, we now define it as an interpretable, information-driven measure of the equivalent number of independent dimensions required to account for the overall pattern of covariation in the data, obtained from the full eigenvalue spectrum. We also state its benefit explicitly: ED condenses the entire spectrum into a single, interpretable index of how many dimensions behavior effectively occupies, with a low value indicating tighter cross-variable coordination.

In the Results, where ED is first applied, we added that reducing the whole spectrum to one comparable number per timepoint is precisely what allows us to track the simplification of behavior continuously and to test it uniformly across all ten critical events.

Finally, in the Methods, we now open the corresponding subsection by framing the squared cosine and ED as two complementary PCA-derived descriptors, and we added a connecting sentence so the two measures read as a coherent progression (per-variable representation followed by the dimensionality of the representation as a whole) rather than as separate items.

Together, these additions make explicit what ED measures and why it is a useful, compact index for our analysis.

Comment 12: In the example figure, participants are seated behind a steering wheel in Level 5 - why is this the case?

Authors' response:

Done – We thank the reviewer for this question. As the reviewer points out, participants remained seated behind the steering wheel in both driving conditions. **We have made important edits in the "experimental setup" to convey that** this was done for two reasons: first, efficiency, as the vehicle mockup comprised two adjacent stations, which allowed us to simultaneously record two participants. Second, since participants under both conditions remained seated behind the steering wheel, we could obtain comparable field of view, facilitating a comparison of visuomotor coordination strategies. Importantly, regardless of the physical station, the VR consistently rendered a left-hand driving scene with the steering wheel on the left. In this sense, our automated condition is more compatible SAE level 4, where the automated system handles all driving tasks with no specific seating restrictions.

Added statement: "The vehicle mockup comprised two adjacent stations [...] The two stations allowed two participants to be tested simultaneously and independently under a randomly assigned manual or automated condition. In both conditions, participants remained seated behind the steering wheel to ensure a consistent field of view, facilitating a valid comparison of visuomotor coordination strategies. Regardless of the physical station, the VR consistently rendered a left-hand driving scene with the steering wheel on the left."

Comment 13: Steering data is only meaningful for manual driving; how is this accounted for? The claim that "Manual driving was characterized by steering-dominant coordination patterns within the low-dimensional space" is unsurprising given that no steering data is available (or carries any behavioral meaning) in automated driving. Additionally, what kind of steering data was used? One would typically expect specific metrics such as steering entropy or steering wheel reversal rate, rather than the raw steering angle itself

Authors' response:

Done/addressed – We thank the reviewer for the comment; **in the revised manuscript we addressed all three points**. First, on the asymmetry and the "unsurprising" manual/steering pattern (points 1–2): we agree. Because the automated drive followed a predetermined trajectory, the steering-wheel angle carried between-participant variance mostly in the manual condition, so a steering-based separation is expected by design. We now state this explicitly and reframe the claim in the Discussion: the informative result is not that steering separates the groups, but that in both groups gaze couples to the direction of the car, actively together with steering in manual driving and passively, in the absence of steering control, in automated driving, where drivers still monitor where the car is heading. This is consistent with visuomotor coordination for steering persisting even when vehicle control is automated (Navarro et al. 2020) (Lines 613–621). We also clarified in the Methods (Segmentation and principal components analysis) that steering was retained because it indexes the hand's angular contribution to eye–head–hand–vehicle coordination and shares the same angular unit as the other signals, whereas longitudinal kinematics were excluded (see Comment: 6).

Second, on the type of steering signal (point 3): **we edited the text to state that** "steering input was recorded in radians from the Fanatec steering wheel device and converted to degrees". Thus, it is clear that we used the steering-wheel angle, not a derived index such as steering entropy or the steering-wheel reversal rate. This choice is deliberate: our time-resolved PCA characterizes the instantaneous angular covariation among effectors (eye, head, vehicle, and hand), for which an angular signal expressed in a common unit is a more appropriate input. Steering entropy and reversal rate instead summarize the temporal regularity of corrective steering movements and are used primarily as markers of attention-related states such as distraction or fatigue, which lie outside the

sensorimotor-coordination question we meant to address here. They would be a valuable direction for a workload- or attention-focused analysis.

Comment 14: Several references to the statistical methods employed are missing from the analysis section

Authors' response:

Done – We thank the reviewer for pointing this out. **We have now added references for the statistical methods employed throughout the analysis section.** Specifically, the Methods now cite the sources for the Pearson correlations relating variables to the principal components (Lê et al., 2008 [79]; Benesty et al., 2009 [81]); the squared cosine quality of representation measure (Abdi & Williams, 2010 [33]; Jolliffe & Cadima, 2016 [35]; Lê et al., 2008 [79]); the chi-square test used to compare the before- and after-onset windows (Press, 2007 [82]); the Benjamini–Hochberg false-discovery-rate correction (Benjamini & Hochberg, 1995 [85]); the stratified k-fold cross-validation and the AUC-ROC used to quantify classifier performance (Kohavi, 1995 [86]; Fawcett, 2006 [87]). We trust that the statistical methods employed are now adequately referenced.

Comment 15: The text in Fig 1 is too small

Authors' response:

Done – We thank the reviewer for the comment. **We have enlarged the text** in Fig 1 (and all other figures) for legibility.

Comment 16: The wording "early components" is somewhat confusing, as "early" implies a temporal dependency. If I understand correctly, it refers to "the first two principal components." If so, I would suggest using that phrasing consistently

Authors' response:

Done – We thank the reviewer for highlighting the confusion the term "early components" can lead to. To disambiguate this, **we updated the text** and use the terms "leading components" and "first two principal components" consistently throughout the text instead of "early components".

Comment 17: It is unclear whether "exceeding 50% of the cumulative variance" is sufficient. What would be gained by adding a third component? Could an ablation be run here?

Authors' response:

Done – We thank the reviewer for this valuable suggestion. To address this concern, **in the revised manuscript, we performed a component-sweep ablation** in which we trained the same stratified K-fold cross-validated logistic regression classifier using a third component. Adding a third component yielded only a marginal improvement in classification performance, confirming that the first two PCs capture enough condition-discriminative information and that the third component contributes little. **This analysis is included in Figure 8C** (included below), which has been updated to display the AUC time courses for all three component sets, showing that PC1-3 behaves very similarly over time as PC1-2. **We have included the corresponding results in the results section** (Lines 504–510).

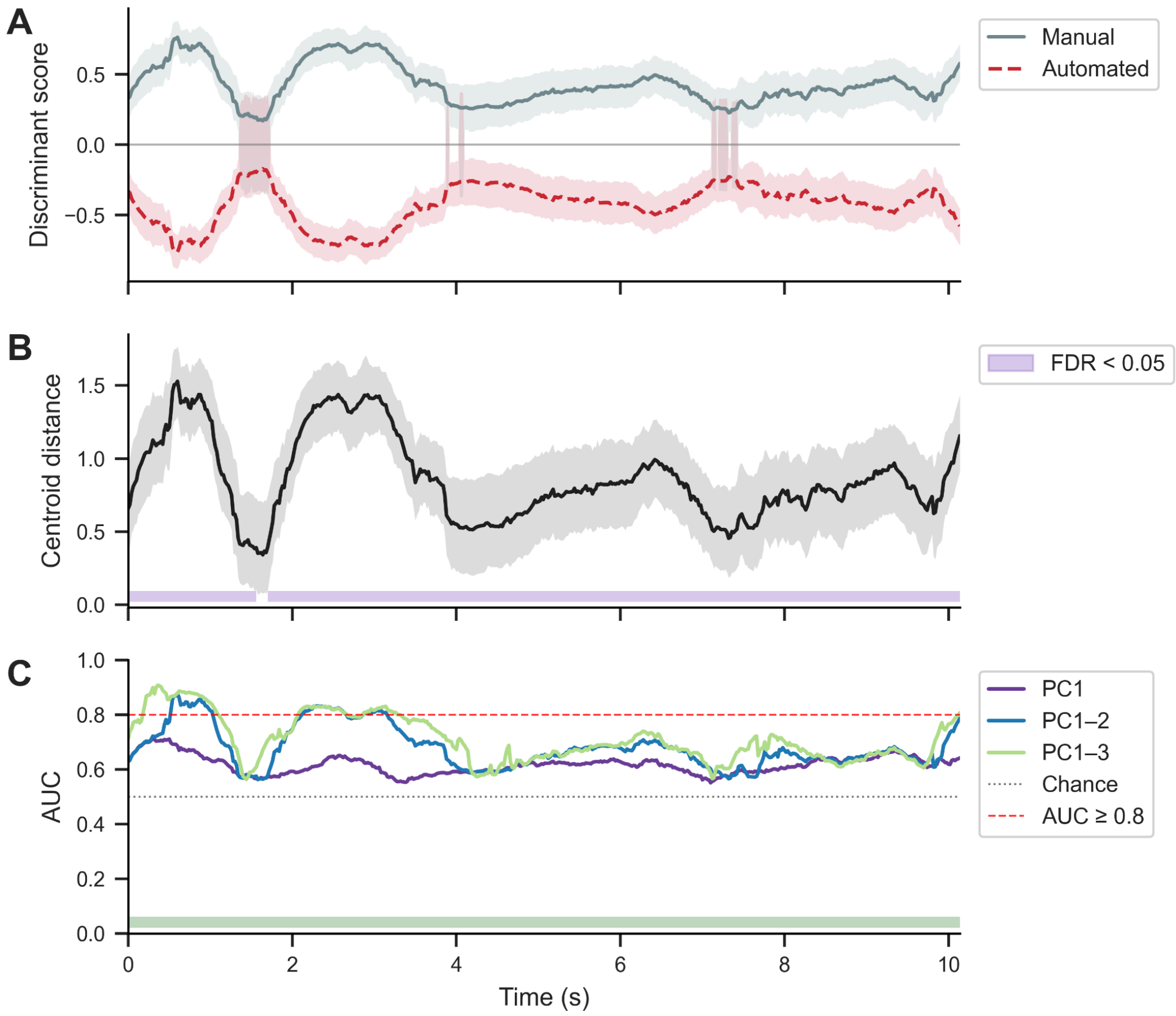

Comment 18: "Driving, for instance, means constantly turning what we see into the right movements of our head, hands, and feet." --> Or do we turn our head to see the things that are most important for achieving our goals?

Authors' response:

Done – We thank the reviewer for this insightful and provocative comment. The reviewer is right that our original phrasing captured only one aspect, as head movements can also guide our gaze. **We have revised the sentence in the Author summary to make the two-way coupling explicit:** "Driving, for instance, involves actively turning our head and eyes to see what matters most while translating those movements into appropriate actions with our hands and feet."

Comment 19: "A second aspect that makes behavior complex concerns its high variability." - Very general sentence that does not add too much value

Authors' response:

Done – We thank the reviewer for this observation. We agree that the sentence was vague and **have replaced the general opening sentence with a more specific and informative one** that both signals the paragraph's topic and states what variability means. The paragraph now begins: "A second source of behavioral complexity is variability, the observation that repeated executions of the same task rarely reproduce identical movements [..]" and we adjusted the following sentence to avoid redundancy.

Comment 20: The intro might benefit from clarifying what the differences between a subspace and manifold are

Authors' response:

Done – We thank the reviewer for this suggestion. **In the revised manuscript**, rather than adding a detailed definition, which would unnecessarily increase the length of the Introduction, we now draw the distinction subtly at first mention, noting that PCA yields linear subspaces and that those subspaces might serve as local building blocks for describing more complex, nonlinear structures such as manifolds (Introduction, third paragraph). This makes the subspace/manifold relationship clear while also signaling that approaches beyond PCA are available, with references provided for interested readers (Mante et al., 2013 [31]; Soldado-Magraner et al., 2024 [32]).

Comment 21: "A transformation of high-dimensional behavioral variables into common and synchronized features underlying the production of skilled behavior [56, 57]." - I don't get this sentence

Authors' response:

Done – We thank the reviewer for pointing this out. The sentence was fragmented, and it was meant to read as part of the previous sentence. **We have rewritten it as a complete sentence and merged it with the preceding one to remove redundancy.** It now reads: "[...] coordinated, skillful action involves overcoming superfluous DoF by transforming high-dimensional behavioral variables into common, synchronized features (i.e., motor synergies) that underlie the production of behavior [58, 59]." (Lines 81-83). This clarifies that the transformation refers to the formation of motor synergies.

Comment 22: There is nothing such as autonomous vehicle operation. It's automated driving classified according to the 5 SAE Levels (See SAEJ3016)

Authors' response:

Done – We thank the reviewer for pointing this out and agree that the suggested wording is more appropriate. Thus, **we have adopted SAE J3016 terminology** and updated the wording using "automated driving" throughout the text and figures, and cited SAE J3016.

Comment 23: "presentation and a wooden car." - Wooden Car sounds very weird. It's a vehicle mock up

Authors' response:

Done – We thank the reviewer for the suggestion. **We have replaced** 'wooden car' with 'vehicle mockup' throughout the manuscript.

Comment 24: A table showing an overview of all variables that were collected would be helpful

Authors' response:

Done / addressed – We thank the reviewer for this helpful suggestion. We agree that a clear overview of the variables improves accessibility. To keep the manuscript concise and avoid over-extending the Methods section with an exhaustive variable-by-variable table, **we have added a pointer to a table openly available in the project's repository.** Specifically, we now state that "A complete overview of all recorded variables and their definitions is provided in the project's repository." This ensures accessibility to readers while keeping the main text focused and readable.

Comment 25: The last part of the section "Segmentation and principal components analysis" starting at line 175 is unclear

Authors' response:

Done – We thank the reviewer for pointing out that this paragraph was unclear. **We have rewritten the final part of the "Segmentation and principal components analysis" and made it clearer.** The revised text now states explicitly what the time-resolved PCA yields at each timepoint (the eigenvalues and associated explained variance, the participants' component scores, and the variable loadings), and how these outputs were used in the subsequent analyses. It also emphasizes that, because a PCA is obtained at every timepoint, analyzing them all to obtain insights into sensorimotor strategies via biplots was impractical, and thus we traced strategic timepoints of low and high ED. Specifically, the event onset and offset, and the after onset peak and subsequent decline in the cumulative variance of the first two PCs. We have edited the paragraph accordingly to improve clarity.

Comment 26: Mahalanobis-distance-based procedure - Not referenced or introduced

Authors' response:

Done – We thank the reviewer for pointing this out. **We have expanded the description in the Data Preprocessing section to clarify that the outlier screening uses a robust variant of the Mahalanobis distance** based on the Minimum Covariance Determinant (MCD) estimator and included the respective references we have used. Unlike the standard Mahalanobis distance, which relies on the sample mean and covariance matrix of the full sample, the MCD estimates location and scatter from the least contaminated subset of observations, making it more robust for detecting outliers.

Comment 27: The description of the gaze data processing is way to verbose. This could easily go into the appendix

Authors' response:

Done – We thank the reviewer for this comment. We agree that the original gaze- and head-processing description was overly verbose. **We have substantially condensed the Data preprocessing subsection by removing redundant narrative**, while retaining the essential conversion steps and the key equations for gaze angles, quaternion localisation, and radian-to-degree conversion.

Because **the manuscript now follows the PLOS section order with Materials and methods placed after the Discussion and Conclusion**, detailed preprocessing is already separated from the main narrative flow and remains readily accessible for readers who wish to inspect the formulas. We therefore kept the condensed description and equations in the Methods section rather than adding a separate appendix.
