# Supplementary material for: Visuomotor coordination on the road: Low-dimensional representations reveal adaptive, context-dependent reductions in the dimensionality of natural driving behavior
Source: PLoS Comput Biol. 2026 Oct 23;22(10):e1014056. doi: 10.1371/journal.pcbi.1014056 (PMC13722334; doi:10.1371/journal.pcbi.1014056)
Supplement: Supplementary file 4 [file pcbi.1014056.s007.pdf]

Response to reviewers' comments

To: Editor and Reviewers

Subject: Responses to Reviewers' Comments for Visuomotor coordination on the road: low-dimensional representations reveal adaptive, context-dependent reductions in the dimensionality of natural driving behavior (PCOMPBIOL-D-26-00403R1) - [EMID:0259ff2bb1ee2b28]

Dear Editor, dear Reviewers,

We thank the editors and both reviewers for their positive assessment of the revised manuscript and for their continued, constructive comments that have led to the improved version submitted here. We are pleased that both reviewers and the editor found the manuscript considerably improved. We also thank the editor for handling the remaining points as a minor revision without requiring further involvement of the reviewers.

Nevertheless, in this revision, we have addressed each of Reviewer 2's remaining comments in detail. We revised the Introduction and the Discussion, extending the limitations and adding a paragraph on the implications of our findings for the design of automated driving systems. We have also implemented the editorial points raised in the Journal Requirements.

Below, we provide a point-by-point response to the remaining comments, detailing how and where each point was addressed. Line numbers refer to the marked-up version of the revised manuscript.

Sincerely,

John Madrid-Carvajal (on behalf of all authors)

Response to Reviewer #1

Comment 1: Thanks to the authors for the thorough response letter and the additional analyses. Personally, I was very happy to see the results regarding the stability of intersubject differences the low-dimensional across events and I think this is a very nice and interesting addition. Overall, through all of the re-writing and tuning of the manuscript, I feel that it is now substantially improved and in my opinion ready for publication.

Authors' response: We thank the reviewer for such positive assessment of the revision; we are glad that the reviewer found the cross-events stability analysis as insightful and interesting as we did. We also appreciate the reviewer's suggestions, which helped us improve the manuscript. No further changes were requested.

Response to Reviewer #2

General: In the following, I will only assess how the authors addressed the comments I made in the first revision:

Authors' response: We thank the reviewer for the assessment of the edits made to the revised manuscript.

Comment 1: The introduction reads much better now.

Comment 2: While I appreciate the formulation of clear questions/objectives, formulating them after the fact is not good scientific practice. In particular, the addition of question one: "behavior that is neither repeated nor experimentally constrained" makes the impression that a former weakness of the study (not explicitly mentioning and describing this) is trying to be turned into a strength. It leaves the impression of HARKing. Still, the introduction reads much better and overall the purpose is clearer.

Authors' response: We thank the reviewer for the positive assessment of the Introduction and for this comment. We understand how the explicit questions could give the impression of having been formulated after the fact. **To avoid this impression, in the revised manuscript, we have rephrased the opening of the final paragraph of the Introduction** (line 90), which now states that the three questions translate the general motivation described in the preceding paragraphs (incl. Author summary) into concrete aims, and we present them as questions rather than as hypotheses. We believe this keeps the clearer statement of purpose the reviewer found helpful, while making explicit that the questions follow from the motivation of the study.

Comment 3: Comment 3: Thank you for the important clarifications. This is much better now.

Authors' response: We thank the reviewer for confirming that the clarifications made in the revision to the experimental setup and procedure resolved this point.

Comment 4: Sorry if I was not clear: by demographics, I was also referring to questions like "Did the participants have a valid driver's license?" I consider this to be a true threat to validity. Imagine half of the participants did not undergo driver training, or it would have been the first time "driving" a car. This would invalidate the whole study. Please elaborate on this.

Authors' response:

Done – We thank the reviewer for this clarification. **In the revised manuscript, we have therefore expanded the limitations in the Discussion** to address this concern explicitly (starting at line 713). There, we acknowledge that, due to incomplete demographic information, the generalizability of our results to licensed drivers remains to be established. We also describe two aspects of the study that mitigate this concern: the standardized training scene all participants completed before the main drive (including two practice events), and participants' general familiarity with traffic through school-based traffic education in Germany.

Comment 5: Fine!

Authors' response: We thank the reviewer for confirming that the edits addressing the simulator-related limitations in the discussion resolved this point.

Comment 7: Thank you, this should also lead to an updated discussion.

Authors' response:

Done – We thank the reviewer and agree. Thus, in the revised manuscript, we have extended the Discussion to state explicitly how the variance results relate to the reorganisation of variable contributions and the decrease in effective dimensionality (starting at line 607).

Comment 8: The new discussion reads much better. However, it is still quite flat when it comes to the "so what?" question in terms of informing the design of automated systems and how these insights can actually improve automated driving systems. Here I would have wished at least for an attempt to connect these theoretical results to actionable choices and system design or the development of predictive models in DMS or similar.

Authors' response:

Done – We thank the reviewer for the positive comment and agree that the "so what" is a fair challenge. In the revised manuscript, we have added a paragraph to the Discussion where we attempt to connect how the low-dimensional behavior results could inform the design of automated driving systems, while keeping this link conceptual (lines 635–653).

Comment 9-11: Fine

Authors' response: We thank the reviewer for confirming that Comments 9-11 have been successfully resolved.

Comment 12: I feel this is much more of a limitation than you describe. You argue that your analysis shows that eye movements still follow the road even though being in automated driving mode. One reason for this might be that sitting behind a steering wheel indicates that a take-over or intervention by the driver might be needed. Therefore, drivers might stay alert. On the other hand if sitting on a passenger seat with no steering wheel. This might change completely as there is not even the possibility for any driving-related intervention. Therefore, the fact that this is rather Level 4 driving and might not at all apply to level 5 should be discussed.

Comment 13: see comment 12 - this goes together

Authors' response:

Done – We thank the reviewer for pointing this out. We agree that because participants in the automated condition remained seated behind the steering wheel, this condition corresponds to SAE level 4 rather than level 5. In the manuscript, we have made this clear and edited the Discussion section accordingly (lines 649–653). We state explicitly that the low-dimensional signatures should not be assumed to hold for SAE level 5 where no human-related attention or intervention is required. In line with Comment 13, the same paragraph notes that steering is by definition absent under the automated condition, so it is characterised by the coupling of gaze with vehicle heading rather than by steering.

Comment 13-15 [incl. 16]: Thank you for addressing these minor changes

Authors' response: We thank the reviewer for confirming that these minor changes have been successfully addressed.

Comment 17: Nice analysis, thanks!

Authors' response: We thank the reviewer for the positive assessment of the additional analysis.

Comment 18 - Comment 27: All fine, thanks!

Authors' response: We thank the reviewer for confirming that Comments 18-27 have been successfully addressed.
