# Supplementary material for: Visuomotor coordination on the road: Low-dimensional representations reveal adaptive, context-dependent reductions in the dimensionality of natural driving behavior
Source: PLoS Comput Biol. 2026 Oct 23;22(10):e1014056. doi: 10.1371/journal.pcbi.1014056 (PMC13722334; doi:10.1371/journal.pcbi.1014056)
Supplement: Peer review history

# Peer review: Decision Letter 0

**DOI:** 10.1371/journal.pcbi.1014056.r001

**Authors:** Thomas Serre, Christoph Strauch

19 May 2026

Visuomotor coordination on the road: low-dimensional representations reveal adaptive, context-dependent reductions in the dimensionality of natural driving behavior

PLOS Computational Biology

Dear Dr. Madrid-Carvajal,

Thank you for submitting your manuscript to PLOS Computational Biology. After careful consideration, we feel that it has merit but does not fully meet PLOS Computational Biology's publication criteria as it currently stands. Therefore, we invite you to submit a revised version of the manuscript that addresses the points raised during the review process.

Please submit your revised manuscript by Jul 19 2026 11:59PM. If you will need more time than this to complete your revisions, please reply to this message or contact the journal office at ploscompbiol@plos.org. When you're ready to submit your revision, log on to https://www.editorialmanager.com/pcompbiol/ and select the 'Submissions Needing Revision' folder to locate your manuscript file.

Please include the following items when submitting your revised manuscript:

* A letter that responds to each point raised by the editor and reviewer(s). You should upload this letter as a separate file labeled 'Response to Reviewers'. This file does not need to include responses to formatting updates and technical items listed in the 'Journal Requirements' section below.

* A marked-up copy of your manuscript that highlights changes made to the original version. You should upload this as a separate file labeled 'Revised Manuscript with Track Changes'.

* An unmarked version of your revised paper without tracked changes. You should upload this as a separate file labeled 'Manuscript'.

We look forward to receiving your revised manuscript.

Kind regards,

Christoph Strauch

Academic Editor

PLOS Computational Biology

Thomas Serre

Section Editor

PLOS Computational Biology

**Additional Editor Comments:**

Two expert reviewers have read the manuscript and provided clear guidance on how to strengthen the manuscript. While we believe that this will require quite some work, we are hopeful that these points can be meaningfully addressed in a revised version of the paper.

**Journal Requirements:**

If the reviewer comments include a recommendation to cite specific previously published works, please review and evaluate these publications to determine whether they are relevant and should be cited. There is no requirement to cite these works unless the editor has indicated otherwise.

1) We ask that a manuscript source file is provided at Revision. Please upload your manuscript file as a .doc, .docx, .rtf or .tex. If you are providing a .tex file, please upload it under the item type u2018LaTeX Source Fileu2019 and leave your .pdf version as the item type u2018Manuscriptu2019.

2) Your manuscript's sections are not in the correct order.  Please amend to the following order: Abstract, Introduction, Results, Discussion, and Methods

3) We notice that your supplementary Tables are included in the manuscript file. Please remove them and upload them with the file type 'Supporting Information'. Please ensure that each Supporting Information file has a legend listed in the manuscript after the references list.

4) 1A includes an image of an identifiable person.

Figure [X] includes an image of an identifiable person. Please confirm that you have obtained written confirmation or release forms, signed by the subject(s) (or their guardian), giving permission to be photographed and to have their images published under a Creative Commons license. You can find more details on the journal's informed consent policy and a downloadable Consent Form for Publication in a PLOS Journal here: journals.plos.org/plosone/s/human-subjects-research#loc-patient-privacy-and-informed-consent-for-publication.

Please do NOT upload this form as part of your submission files, in order to protect the privacy of the patient's identity. Instead, in the Ethics Statement in the Methods section of your manuscript, please confirm that the patient has provided consent by inserting a statement such as the following:

The individual in this photograph has given written informed consent (as outlined in PLOS consent form) to publish this image.

If you have not received consent from the photograph subject to publish their image, please remove the image or blur or crop the individual in the photo so that their face is not visible.

5) Some material included in your submission may be copyrighted. According to PLOS’s copyright policy, authors who use figures or other material (e.g., graphics, clipart, maps) from another author or copyright holder must demonstrate or obtain permission to publish this material under the Creative Commons Attribution 4.0 International (CC BY 4.0) License used by PLOS journals. Please closely review the details of PLOS’s copyright requirements here: PLOS Licenses and Copyright. If you need to request permissions from a copyright holder, you may use PLOS's Copyright Content Permission form.

Please respond directly to this email and provide any known details concerning your material's license terms and permissions required for reuse, even if you have not yet obtained copyright permissions or are unsure of your material's copyright compatibility. Once you have responded and addressed all other outstanding technical requirements, you may resubmit your manuscript within Editorial Manager.

Potential Copyright Issues:

i) Please confirm (a) that you are the photographer of 1A, or (b) provide written permission from the photographer to publish the photo(s) under our CC BY 4.0 license.

ii) Figures 1B, 1C, and 3. Please confirm whether you drew the images / clip-art within the figure panels by hand. If you did not draw the images, please provide (a) a link to the source of the images or icons and their license / terms of use; or (b) written permission from the copyright holder to publish the images or icons under our CC BY 4.0 license. Alternatively, you may replace the images with open source alternatives. See these open source resources you may use to replace images / clip-art:

- https://commons.wikimedia.org

- https://openclipart.org/.

6) Please amend your detailed Financial Disclosure statement. This is published with the article. It must therefore be completed in full sentences and contain the exact wording you wish to be published.

1) State the initials, alongside each funding source, of each author to receive each grant. For example: "This work was supported by the National Institutes of Health (####### to AM; ###### to CJ) and the National Science Foundation (###### to AM).".

If you did not receive any funding for this study, please simply state: u201cThe authors received no specific funding for this work.u201d

7) Please send a completed 'Competing Interests' statement, including any COIs declared by your co-authors. If you have no competing interests to declare, please state "The authors have declared that no competing interests exist". Otherwise please declare all competing interests beginning with the statement "I have read the journal's policy and the authors of this manuscript have the following competing interests"

**Reviewers' comments:**

Reviewer's Responses to Questions

**Comments to the Authors:**

**Please note here if the review is uploaded as an attachment.**

Reviewer #1: In their study Madrid Carvajal and colleagues analyze a large dataset of people in a driving simulator in virtual reality. Participants passed a series of different small events and the dimensionality of behavior was assessed. Participants either completed a manual session, where they were in control of the car, or an automated session, where people only were sitting in the car while steering was performed automatically. The main result was that only two principal components could explain a large proportion of variance across eye, head and vehicle behavior, and this dimensionality decreased even further around the time of the critical events. With these factors it was also possible to discriminate the manual from the autonomous conditions.

Overall, the manuscript is well written, the data are interesting and complex (and freely available), and the analysis is an interesting approach. I have mainly some comments with respect to the analysis.

Methods: The authors performed an alignment of the data to a standard time. While I understand the necessity of this step for the future analysis, could this alignment hide some of the inter-participant variability? For example, some people might go overall faster and some overall slower, and as far as I see, variables like acceleration of the car were measured but are not included in the PCA. Would adding the average car speed change something in the main results?

Methods: Out of interest, did people suffer from motion sickness in the simulator? Could this explain some types of the observed behavior?

Results: If I understand correctly, since a large part of the analysis is focused on 1 particular event, it would be really helpful to describe the event in question a bit in detail. This will help with the interpretation and understanding of the data.

Results: One pattern that seems quite clear in Fig 7, but can also be seen in the other traces: The reduction in the effective dimensionality often starts before 0, so it is not really a sharp drop after the event, but it seems to be a predictive response that shows a preparation to respond to a future event. One could try to test whether that pattern is consistent (so when is the onset of the drop) and whether this timing differs across events, and is related to at which point in time something is visble (e.g. the merging of a lane can be seen from further away than an animal suddenly stepping on the street).

Results: Potentially additional interesting analysis: When you have the subspace of minimal behavior for one event, you get the similarity of subjects in that space for one event. Is this scaling comparable to how similar people where in response to other events? Or in other words, is how people behave in the low-dimensional space stable?

Results: Is there any way to link the behavior to actual performance? I guess this would be only interesting for the manual condition, but is the low-dimensional representation predictive of some objective performance markers, e.g. the smoothness of the steering, Amount of breaking or minimum distance to an obstacle?

Minor Comments

Line 117: trajectories of eye, head and car along with their positions? Isn’t the trajectory made out of the different positional measurements? That sentence is a bit unclear to me.

Line 153: How are valid eye tracking data defined? Was there some custom calibration?

Line 163: Within a time segment, did you average the data for the individual measurements (e.g. average eye vertical position?) Please clarify.

Line 272ff: The abbreviations CV and FDR are introduced, but not explained. It might be helpful to do so.

Figure 6: In the PDF version, the quality of the Figure is unfortunately not great, it is very hard to see especially the smaller labels and numbers in Panels B & C. Please make sure, that the quality is good enough. One way to improve the figure could potentially is to only highlight the first two figures in Panel B & C, since the other two are not discussed in the text. Also in Panel A, it might be helpful to align the time axis to 0 at event onset.

Figure 8: Again, it could potentially be helpful to change the time axis with 0 as event onset

Reviewer #2: Points in Favor

The large number or participants is very positive, in particular considering that this is a simulator study. Using and open exhibition space to collect the data is a very nice idea. Even though wth some drawbacks (see my later comments)

Overall, I like the idea of the approach and the insights that are generated even though they are mostly of theoretical interest

The writing is adequate, but

The figures are informative and are a valuable extension to the text. The overall visual appearance is good

I appreciate the effort that the authors put into the OSF project and overall documentation and that they share their data, code and other artifacts

Major Issues

The structure of the introduction needs improvement. Currently, it alternates between motivation, methods, and related work, which makes it very hard to follow and confuses the reader. Furthermore, the driving use case is introduced very late. It is in the title, yet the reader has to get through several pages before it is first mentioned.

No research questions are formulated, even though hypothesis testing is performed. Why is that? Positioning this work as a clearly explanatory study would help

The study set-up is not really clear: How controlled were the surroundings (sound, etc.) given that the study was conducted in an open exhibition space? Was an experimenter present at all times? How were participants briefed? There are a lot of open questions here. Furthermore in the picture in Fig1 the steering wheel is on the right in the physical simulator but on the left in the VR simulation. How does this go together? Aslo why are there two people with hedsets in the simulator. Was this the study set-up?

No demographic information is provided, which makes it hard to assess the comparability of the two groups and is a serious threat to validity that should be discussed in the limitations section in the discussion. It is also not clear if participants were able to choose if they want to perform the automated or manual drive. This might introduce a bias between the groups

Given the videos that were attached it becomes obvious that the physics and driving behavior of the automated car are somewhat unrealistic. Simulator-induced limitations should be mentioned in the discussion

The statistical analysis needs clarification. It is very hard to follow why specific decisions were made (comparing BO vs. AO, the ED abbrevaton is not introduced on first use, why did the authors choose a 5 second window size?). The choice of statistical tests is also not properly explained, for instance, why was an inverse-variance-weighted chi-square test used? Overall, I had to re-read this section multiple times and am still unsure why the analysis is designed in exactly this way. The paper would benefit from a clearer conceptual explanation of the overarching approach

"The PCs' variance distributions across all events show a clear variance redistribution" — I do not agree. PC2–PC5 are nearly indistinguishable, and the insight one can draw from this seems negligible in practice due to the extremely small effect size

The connection between the many analyses performed and the actual, concrete learnings about driver behavior is often unclear. In the discussion, the authors state that "Together, these findings indicate that the visuomotor coordination underlying driving behavior is both low-dimensional and context-sensitive, with hazard-related compression revealing group-specific coordination patterns." A provocative but fair question here is: "So what?" Stronger contextualization within the driving literature — and a clearer account of how these results could improve our understanding of how people drive, or even inform better automated systems — would give the paper considerably more leverage

Minor Comments and Suggestions

The section structure is somewhat unusual, especially in the Materials and Methods, where the analysis is discussed before the processing, and the ethics statement appears in the middle

The very detailed description of what PCA is in the introduction seems misplaced. In particular, if one considers how many statistical concepts and methods are not introduced in the analysis part of the paper

Effective dimensionality is introduced without explaining what it is, or why knowing it is necessary or beneficial

In the example figure, participants are seated behind a steering wheel in Level 5 — why is this the case?

Steering data is only meaningful for manual driving; how is this accounted for? The claim that "Manual driving was characterized by steering-dominant coordination patterns within the low-dimensional space" is unsurprising given that no steering data is available (or carries any behavioral meaning) in automated driving. Additionally, what kind of steering data was used? One would typically expect specific metrics such as steering entropy or steering wheel reversal rate, rather than the raw steering angle itself

Several references to the statistical methods employed are missing from the analysis section

The text in Fig 1 is too small

The wording "early components" is somewhat confusing, as "early" implies a temporal dependency. If I understand correctly, it refers to "the first two principal components." If so, I would suggest using that phrasing consistently

It is unclear whether "exceeding 50% of the cumulative variance" is sufficient. What would be gained by adding a third component? Could an ablation be run here?

"Driving, for instance, means constantly turning what we see into the right movements of our head, hands, and feet." --> Or do we turn our head to see the things that are most important for achieving our goals?

"A second aspect that makes behavior complex concerns its high variability." - Very general sentence that does not add too much value

The intro might benefit from clarifying what the differences between a subspace and manifold are

"A transformation of 79 high-dimensional behavioral variables into common and synchronized features 80 underlying the production of skilled behavior [56, 57]."  - I don't get this sentence

There is nothing such as autonomous vehicle operation. It's automated driving classified according to the 5 SAE Levels (See SAEJ3016)

"presentation and a wooden car." - Wooden Car sounds very weird. It's a vehicle mock up

A table showing an overview of all variables that were collected would be helpful

The last part of the section "Segmentation and principal components analysis" starting at line 175 is unclear

Mahalanobis-distance-based procedure - Not referenced or introduced

The description of the gaze data processing is way to verbose. This could easily go into the appendix

**********

**Have the authors made all data and (if applicable) computational code underlying the findings in their manuscript fully available?**

The PLOS Data policy requires authors to make all data and code underlying the findings described in their manuscript fully available without restriction, with rare exception (please refer to the Data Availability Statement in the manuscript PDF file). The data and code should be provided as part of the manuscript or its supporting information, or deposited to a public repository. For example, in addition to summary statistics, the data points behind means, medians and variance measures should be available. If there are restrictions on publicly sharing data or code —e.g. participant privacy or use of data from a third party—those must be specified.

Reviewer #1: Yes

Reviewer #2: Yes

**********

PLOS authors have the option to publish the peer review history of their article (what does this mean?). If published, this will include your full peer review and any attached files.

If you choose “no”, your identity will remain anonymous but your review may still be made public.

**Do you want your identity to be public for this peer review?**  For information about this choice, including consent withdrawal, please see our Privacy Policy.

Reviewer #1: No

Reviewer #2: No

**Figure resubmission:**

**Reproducibility:**

-->?>

# Author comment: Author response to Decision Letter 1

**DOI:** 10.1371/journal.pcbi.1014056.r002

9 Aug 2026

Attachment Submitted filename: Response_to_Reviewers.pdf

# Peer review: Decision Letter 1

**DOI:** 10.1371/journal.pcbi.1014056.r003

**Authors:** Thomas Serre, Christoph Strauch

15 Sep 2026

PCOMPBIOL-D-26-00403R1

Visuomotor coordination on the road: low-dimensional representations reveal adaptive, context-dependent reductions in the dimensionality of natural driving behavior

PLOS Computational Biology

Dear Dr. Madrid-Carvajal,

Thank you for submitting your manuscript to PLOS Computational Biology. After careful consideration, we feel that it has merit but does not fully meet PLOS Computational Biology's publication criteria as it currently stands. Therefore, we invite you to submit a revised version of the manuscript that addresses the points raised during the review process.

Please submit your revised manuscript by Nov 15 2026 11:59PM. If you will need more time than this to complete your revisions, please reply to this message or contact the journal office at ploscompbiol@plos.org. When you're ready to submit your revision, log on to https://www.editorialmanager.com/pcompbiol/ and select the 'Submissions Needing Revision' folder to locate your manuscript file.

Please include the following items when submitting your revised manuscript:

* A letter that responds to each point raised by the editor and reviewer(s). You should upload this letter as a separate file labeled 'Response to Reviewers'. This file does not need to include responses to formatting updates and technical items listed in the 'Journal Requirements' section below.

* A marked-up copy of your manuscript that highlights changes made to the original version. You should upload this as a separate file labeled 'Revised Manuscript with Track Changes'.

* An unmarked version of your revised paper without tracked changes. You should upload this as a separate file labeled 'Manuscript'.

If you would like to make changes to your financial disclosure, competing interests statement, or data availability statement, please make these updates within the submission form at the time of resubmission. Guidelines for resubmitting your figure files are available below the reviewer comments at the end of this letter.

As the corresponding author, your ORCID iD is verified in the submission system and will appear in the published article. PLOS supports the use of ORCID, and we encourage all coauthors to register for an ORCID iD and use it as well. Please encourage your coauthors to verify their ORCID iD within the submission system before final acceptance, as unverified ORCID iDs will not appear in the published article. Only the individual author can complete the verification step; PLOS staff cannot verify ORCID iDs on behalf of authors.

We look forward to receiving your revised manuscript.

Kind regards,

Christoph Strauch

Academic Editor

PLOS Computational Biology

Thomas Serre

Section Editor

PLOS Computational Biology

**Additional Editor Comments (if provided):**

Both reviewers and myself agree that the manuscript has improved considerably. The suggestions by Reviewer 2 can be addressed by the authors in a minor revision and I do not need to bother the reviewers again for this step.

**Journal Requirements:**

If the reviewer comments include a recommendation to cite specific previously published works, please review and evaluate these publications to determine whether they are relevant and should be cited. There is no requirement to cite these works unless the editor has indicated otherwise.

1) We do not publish any copyright or trademark symbols that usually accompany proprietary names, eg ©,  ®, or TM  (e.g. next to drug or reagent names). Therefore please remove all instances of trademark/copyright symbols throughout the text, including:

- TM on page: 19.

**Reviewers' comments:**

Reviewer's Responses to Questions

**Comments to the Authors:**

**Please note here if the review is uploaded as an attachment.**

Reviewer #1: Thanks to the authors for the thorough response letter and the additional analyses. Personally, I was very happy to see the results regarding the stability of intersubject differences the low-dimensional across events and I think this is a very nice and interesting addition. Overall, through all of the re-writing and tuning of the manuscrip, I feel that it is now substantially improved and in my oppinion ready for publication.

Reviewer #2: In the following, I will only assess how the authors addressed the comments I made in the first revision:

Comment 1: The introduction reads much better now.

Comment 2: While I appreciate the formulation of clear questions/objectives, formulating them after the fact is not good scientific practice. In particular, the addition of question one: "behavior that is  neither repeated nor experimentally constrained" makes the impression that a former weakness of the study (not explicitly mentioning and describing this) is trying to be turned into a strength. It leaves the impression of HARKing. Still, the introduction reads much better and overall the purpose is clearer.

Comment 3: Thank you for the important clarifications. This is much better now.

Comment 4: Sorry if I was not clear: by demographics, I was also referring to questions like "Did the participants have a valid driver's license?" I consider this to be a true threat to validity. Imagine half of the participants did not undergo driver training, or it would have been the first time "driving" a car. This would invalidate the whole study. Please elaborate on this.

Comment 5: Fine!

Comment 6:

Comment 7: Thank you, this should also lead to an updated discussion.

Comment 8: The new discussion reads much better. However, it is still quite flat when it comes to the "so what?" question in terms of informing the design of automated systems and how these insights can actually improve automated driving systems. Here I would have wished at least for an attempt to connect these theoretical results to actionable choices and system design or the development of predictive models in DMS or similar.

Comment 9-11: Fine

Comment 12: I feel this is much more of a limitation than you describe. You argue that your analysis shows that eye movements still follow the road even though being in automated driving mode. One reason for this might be that sitting behind a steering wheel indicates that a take-over or intervention by the driver might be needed. Therefore, drivers might stay alert. On the other hand if sitting on a passenger seat with no steering wheel. This might change completely as there is not even the possibility for any driving-related intervention. Therefore, the fact that this is rather Level 4 driving and might not at all apply to level 5 should be discussed.

Comment 13: see comment 12 - this goes together

Comment 13-15: Thank you for addressing these minor changes

Comment 17: Nice analysis, thanks!

Comment 18 - Comment 27: All fine, thanks!

**********

**Have the authors made all data and (if applicable) computational code underlying the findings in their manuscript fully available?**

The PLOS Data policy requires authors to make all data and code underlying the findings described in their manuscript fully available without restriction, with rare exception (please refer to the Data Availability Statement in the manuscript PDF file). The data and code should be provided as part of the manuscript or its supporting information, or deposited to a public repository. For example, in addition to summary statistics, the data points behind means, medians and variance measures should be available. If there are restrictions on publicly sharing data or code —e.g. participant privacy or use of data from a third party—those must be specified.

Reviewer #1: Yes

Reviewer #2: Yes

**********

PLOS authors have the option to publish the peer review history of their article (what does this mean?). If published, this will include your full peer review and any attached files.

If you choose “no”, your identity will remain anonymous but your review may still be made public.

**Do you want your identity to be public for this peer review?**  For information about this choice, including consent withdrawal, please see our Privacy Policy.

Reviewer #1: No

Reviewer #2: No

[NOTE: If reviewer comments were submitted as an attachment file, they will be attached to this email and accessible via the submission site. Please log into your account, locate the manuscript record, and check for the action link "View Attachments". If this link does not appear, there are no attachment files.]

**Figure resubmission:**

**Reproducibility:**

To enhance the reproducibility of your results, we recommend that authors of applicable studies deposit laboratory protocols in protocols.io, where a protocol can be assigned its own identifier (DOI) such that it can be cited independently in the future. Additionally, PLOS ONE offers an option to publish peer-reviewed clinical study protocols. Read more information on sharing protocols at https://plos.org/protocols?utm_medium=editorial-email&utm_source=authorletters&utm_campaign=protocols

# Author comment: Author response to Decision Letter 2

**DOI:** 10.1371/journal.pcbi.1014056.r004

4 Oct 2026

Attachment Submitted filename: Response to Reviewers.pdf

# Editor report: Decision Letter 2

**DOI:** 10.1371/journal.pcbi.1014056.r005

**Authors:** Thomas Serre, Christoph Strauch

5 Oct 2026

Dear Madrid-Carvajal,

We are pleased to inform you that your manuscript 'Visuomotor coordination on the road: low-dimensional representations reveal adaptive, context-dependent reductions in the dimensionality of natural driving behavior' has been provisionally accepted for publication in PLOS Computational Biology.

Before your manuscript can be formally accepted you will need to complete some formatting changes, which you will receive in a follow up email. A member of our team will be in touch with a set of requests.

Please note that your manuscript will not be scheduled for publication until you have made the required changes, so a swift response is appreciated.

IMPORTANT: The editorial review process is now complete. PLOS will only permit corrections to spelling, formatting or significant scientific errors from this point onwards. Requests for major changes, or any which affect the scientific understanding of your work, will cause delays to the publication date of your manuscript.

Should you, your institution's press office or the journal office choose to press release your paper, you will automatically be opted out of early publication. We ask that you notify us now if you or your institution is planning to press release the article. All press must be co-ordinated with PLOS.

Thank you again for supporting Open Access publishing; we are looking forward to publishing your work in PLOS Computational Biology.

Best regards,

Christoph Strauch

Academic Editor

PLOS Computational Biology

Daniel Bush

Section Editor

PLOS Computational Biology

***********************************************************

Congratulations on this fine manuscript and thanks for taking the last comments seriously and implementing the according changes. I'm looking forward to see the article online!

# Editor report: Acceptance letter

**DOI:** 10.1371/journal.pcbi.1014056.r006

**Authors:** Thomas Serre, Christoph Strauch

PCOMPBIOL-D-26-00403R2

Visuomotor coordination on the road: low-dimensional representations reveal adaptive, context-dependent reductions in the dimensionality of natural driving behavior

Dear Dr Madrid-Carvajal,

I am pleased to inform you that your manuscript has been formally accepted for publication in PLOS Computational Biology. Your manuscript is now with our production department and you will be notified of the publication date in due course.

The corresponding author will soon be receiving a typeset proof for review, to ensure errors have not been introduced during production. Please review the PDF proof of your manuscript carefully, as this is the last chance to correct any errors. Please note that major changes, or those which affect the scientific understanding of the work, will likely cause delays to the publication date of your manuscript.

Soon after your final files are uploaded, unless you have opted out, the early version of your manuscript will be published online. The date of the early version will be your article's publication date. The final article will be published to the same URL, and all versions of the paper will be accessible to readers.

For Research, Software, and Methods articles, you will receive an invoice from PLOS for your publication fee after your manuscript has reached the completed accept phase. If you receive an email requesting payment before acceptance or for any other service, this may be a phishing scheme. Learn how to identify phishing emails and protect your accounts at https://explore.plos.org/phishing.

Thank you again for supporting PLOS Computational Biology and open-access publishing. We are looking forward to publishing your work!

With kind regards,

Janani Seenivasan

PLOS Computational Biology | Carlyle House, Carlyle Road, Cambridge CB4 3DN | United Kingdom ploscompbiol@plos.org | Phone +44 (0) 1223-442824 | ploscompbiol.org | @PLOSCompBiol
